# Desferrioxamine Supports Metabolic Function in Primary Human Macrophages Infected With *Mycobacterium tuberculosis*

**DOI:** 10.3389/fimmu.2020.00836

**Published:** 2020-05-13

**Authors:** James Joseph Phelan, Kate McQuaid, Colin Kenny, Karl Michael Gogan, Dónal J. Cox, Sharee Ann Basdeo, Seónadh O’Leary, Simone Christa Tazoll, Cilian Ó Maoldomhnaigh, Mary P. O’Sullivan, Luke A. O’Neill, Maureen J. O’Sullivan, Joseph Keane

**Affiliations:** ^1^TB Immunology Group, Department of Clinical Medicine, Trinity Translational Medicine Institute, Trinity College Dublin, Dublin, Ireland; ^2^National Children’s Research Centre, Our Lady’s Children’s Hospital, Dublin, Ireland; ^3^School of Biochemistry and Immunology, Trinity Biomedical Science Institute, Trinity College Dublin, Dublin, Ireland

**Keywords:** glycolysis, immunometabolism, Warburg effect, tuberculosis, iron chelation, iron metabolism, HIF1α

## Abstract

Tuberculosis is the single biggest infectious killer in the world and presents a major global health challenge. Antimicrobial therapy requires many months of multiple drugs and incidences of drug resistant tuberculosis continues to rise. Consequently, research is now focused on the development of therapies to support the function of infected immune cells. HIF1α-mediated induction of aerobic glycolysis is integral to the host macrophage response during infection with Mtb, as this promotes bacillary clearance. Some iron chelators have been shown to modulate cellular metabolism through the regulation of HIF1α. We examined if the iron chelator, desferrioxamine (DFX), could support the function of primary human macrophages infected with Mtb. Using RT-PCR, we found that DFX promoted the expression of key glycolytic enzymes in Mtb-infected primary human MDMs and human alveolar macrophages. Using Seahorse technology, we demonstrate that DFX enhances glycolytic metabolism in Mtb-stimulated human MDMs, while helping to enhance glycolysis during mitochondrial distress. Furthermore, the effect of DFX on glycolysis was not limited to Mtb infection as DFX also boosted glycolytic metabolism in uninfected and LPS-stimulated cells. DFX also supports innate immune function by inducing IL1β production in human macrophages during early infection with Mtb and upon stimulation with LPS. Moreover, using hypoxia, Western blot and ChIP-qPCR analyses, we show that DFX modulates IL1β levels in these cells in a HIF1α-mediated manner. Collectively, our data suggests that DFX exhibits potential to enhance immunometabolic responses and augment host immune function during early Mtb infection, in selected clinical settings.

## Introduction

Tuberculosis, caused by *Mycobacterium tuberculosis* (Mtb), is the leading cause of death by a single infectious agent worldwide, killing approximately 1.7 million individuals in 2018 (1, 2). The rise in bacterial resistance to antimicrobial treatment has increased morbidity and mortality among affected patients globally. Host-directed therapy (HDT) is an emerging approach to combat infectious diseases, which aims to support the function of infected host cells (3). HDT predominantly aims to diminish damaging inflammation, however, in early bacterial infections, HDT may enhance antimicrobial properties of infected host immune cells to perturb the dissemination, progression and development of active disease (4–7). As immunometabolic reprogramming is integral to host defense during bacterial infection, supporting these bioenergetic pathways through the use of HDTs may offer alternative therapeutic approaches (1). A shift from oxidative phosphorylation (OXPHOS) to glycolysis has been observed upon infection with Mtb (8, 9). Infection of human macrophages with Mtb is also associated with increased extracellular lactate levels (10). This shift to aerobic glycolysis during Mtb infection is coupled to the ability of human macrophages to produce specific pro-inflammatory cytokines, such as mature IL1β, to lower the burden of infection (10). Pharmacological abrogation of glycolysis in Mtb-infected macrophages also reveals the important role that glycolysis plays in host defense during early Mtb infection (10, 11). *In vivo* studies also highlight the crucial roles of IL1β and TNFα during the early stages of tuberculosis infection (12, 13).

One approach to enhancing glycolysis and immune function is through the modulation of the transcription factor hypoxia-inducible factor 1α (HIF1α). HIF1α is a crucial molecular mediator during Mtb infection (14–16). Importantly, HIF1α is central to reprogramming metabolism toward using aerobic glycolysis and participates in the induction of glycolysis during Mtb infection (1, 14). The stabilization and activity of HIF1α is regulated by the family of prolyl hydroxylase domain (PHD) proteins (17). PHD proteins require iron, α-ketoglutarate (αKG), oxygen and ascorbate in order to inhibit HIF1α. Therefore, the regulation of HIF1α by PHD proteins is tightly coupled with iron availability (1, 18). We previously hypothesized that therapeutic chelation of iron may inhibit PHD protein activity and promote HIF1α stabilization (1). This stabilization of HIF1α may in turn support infected host cells through enhanced glycolysis and increased effector functions.

Iron chelators are administered for the treatment of conditions such as hereditary hemochromatosis and thalassemia (19). Recent studies have also demonstrated therapeutic potential for iron chelators in murine models of pulmonary disease (20). The iron chelators deferiprone, Apo6619 and VK28 all exhibit direct bactericidal activity against *Staphylococcus aureus* and *Escherichia coli* (21). The iron chelators DFX and silybin also reduce Mtb viability in THP-1 monocytes (22). Likewise, iron overload promotes Mtb dissemination in macrophages (23). In agreement with these observations, iron overload was also shown to exacerbate Mtb infection in mice as Mtb also requires iron for survival and competes with the infected host cell for the same iron pool (24, 25). Therefore, we hypothesize that therapeutic iron chelation has potential as a HDT with dual mechanistic activities; by boosting host immunometabolism and immune function through the stabilization of HIF1α. The aim of the current study was to examine if iron chelation enhances immunometabolic profiles and supports host cell function in a human macrophage model of Mtb infection.

## Materials and Methods

### hMDM Cell Culture

PBMCs were isolated from peripheral blood buffy coats (obtained from the Irish Blood Transfusion Services in Dublin, Ireland) by density gradient centrifugation with Lymphoprep^TM^ (Stemcell Technologies). PBMCs were seeded at 2.5 × 10^6^ cells/mL in Roswell Park Memorial Institute (RPMI) 1640 medium (Bio-Sciences), supplemented with 10% AB-human serum (Sigma-Aldrich) and plated onto non-treated tissue culture plates (Corning). LabTeks^TM^ (Nunc) were also seeded to determine the multiplicity of infection (MOI) (see section “Infection of hMDMs and hAMs”). To obtain hMDMs, the cells were differentiated and cultured over 7–10 days at 37°C and 5% CO_2_ prior to experimentation. Non-adherent cells were removed by washing the cells every 2–3 days. The purity of the hMDMs was routinely >95%, as assessed by flow cytometry. For hypoxia analyses, where applicable, hMDMs were cultured under normoxic (21% oxygen) and hypoxic (0.5% oxygen) conditions for 24 h in a Don Whitley *H35 Hypoxystation* unit. hMDMs were exposed to hypoxia from 3 h post infection to ensure low oxygen levels did not affect Mtb uptake.

### hAM Cell Culture

Subsequent to approval by the St. James’s Hospital/Tallaght University Hospital Joint Research Ethics Committee, hAMs were retrieved at bronchoscopy. All donors were patients undergoing clinically indicated bronchoscopy and written informed consent for retrieving additional bronchial washings for research was obtained from all participants prior to the procedure, without subject remuneration. Exclusion criteria included: individuals under 18 years of age, inability to provide written informed consent, any previous or ensuing diagnosis of lung malignancy, sarcoidosis, HIV or Hepatitis C. Patients undergoing biopsy as part of bronchoscopy were also excluded. For the subjects recruited to this study, two individuals were non-smokers and two were ex-smokers. Non-smokers were classified as those subjects reporting no smoking history and ex-smokers were classified as those having ceased smoking more than 6 months prior to sampling. For hAM acquisition, donors were subjected to conscious sedation using intravenous midazolam (lignocaine gel was administered to the nostril). Flexible video-bronchoscope was inserted through the nostril and advanced to the level of the vocal cords by posterior approach, with further lignocaine spray being administered prior to and subsequent to traversing the vocal cords. Following routine bronchoscopy, the bronchoscope was wedged in the right middle lobe bronchus. A total of 180 mL of sterile saline was administered as 60 mL boluses via a connector inserted into the bronchoscope and aspirated within 5 to 10 s under low suction. The procedure was completed in less than 15 min. The bronchoalveolar lavage (BAL) samples were then transported directly to the laboratory for hAM isolation. BAL fluid was filtered through a 100 μm cell strainer (Thermo Fisher Scientific) and centrifuged at 290 *g* for 10 min. The cell pellet was resuspended in 1–2 mL complete RPMI 1640 medium [supplemented with 10% fetal bovine serum (Gibco), 2.7 μM amphotericin B (Fungizone; Gibco) and 110 μM cefotaxime (Melford Biolaboratories)]. Cells were counted using a hemocytometer and plated in 12-well culture plates at a density of 5 × 10^5^ cells/mL. Cell counts from BAL fluid samples ranged between 1.34 × 10^6^ hAMs/mL and 3.5 × 10^6^ hAMs/mL (all resuspended in 1 mL of complete RPMI).

### Mycobacterial Stocks and Culture

Strains of avirulent Mtb H37Ra or virulent Mtb H37Rv were obtained from the American Type Culture Collection (ATCC). Irradiated H37Rv (iH37Rv) was obtained through BEI Resources (NIAID, NIH: Mycobacterium tuberculosis, Strain H37Rv, Gamma-Irradiated Whole Cells, NR-14819). Mtb H37Ra and Mtb H37Rv stocks were propagated in Middlebrook 7H9 broth (Becton Dickinson), made up in low endotoxin WFI H_2_O (Merck Millipore) and supplemented with ADC (albumin, dextrose, catalase) (Becton Dickinson) and 0.05% Tween 80 (Sparks Lab Supplies). Aliquots were stored at −80°C, thawed and propagated in Middlebrook 7H9 broth to log phase prior to use.

### Infection of hMDMs and hAMs

On the day of infection, log phase Mtb was centrifuged at 3000 *g* for 10 min and resuspended in RPMI 1640 medium (supplemented with 10% AB-human serum). The suspension was passed 10 times through a 25 gauge needle and centrifuged at 100 *g* for 3 min to remove any bacterial clumps. The volume of bacterial suspension required for a given MOI was determined by treating macrophages with a range of volumes of resuspended Mtb. hMDMs and hAMs were incubated with Mtb iH37Rv, Mtb H37Ra or Mtb H37Rv for 3 h, washed with pre-warmed PBS to remove extracellular bacteria and fixed with 2% paraformaldehyde (PFA) (Sigma-Aldrich) for 10 min. hMDMs and hAMs were subsequently stained with Modified Auramine-O stain and Modified Auromine-O decolorizer (Scientific Device Laboratory) followed by Hoechst 33242 (Sigma-Aldrich) to counterstain the nuclei. The cells were analyzed under an inverted fluorescent microscope (Olympus IX51) to determine the average number of phagocytosed bacilli per cell and percentage of cells infected. The required volume of bacilli was determined, phagocytic variation was adjusted for between donors to ensure the same MOI (1–10 bacilli/cell, 70% positivity approximately) and the calculated volume of resuspended Mtb added to the appropriate experimental wells. 3 h later, extracellular bacteria were washed off with PBS, fresh complete RPMI added, DFX (Sigma-Aldrich) added (100 μM, in H_2_O) and macrophages were incubated for a further 21 h (24 h total). Non-treated and DFX-treated uninfected hMDMs and hAMs were also assayed in parallel. Un-treated/stimulated, DFX-treated, and lipopolysaccharide (LPS)-stimulated hMDMs were also assayed in parallel as controls (LPS: 100 ng/mL).

### Estimating Cell Number and Cell Viability Using Crystal Violet and Propidium Iodide (PI) Based Cell Exclusion Assays

The crystal violet assay has been previously used to normalize Seahorse data to cell number (26, 27). Upon completion of an experiment, hMDMs were fixed with 1% glutaraldehyde (in PBS) for 15 min at room temperature and washed with PBS. hMDMs were then incubated at room temperature for 20 min with 0.1% crystal violet solution (in dH_2_O). Cells were then washed with dH_2_O and air dried overnight. 1% Triton-X solution (in PBS) was added and the plate gently agitated for 15 min. The solution was then transferred to a 96-well plate, read at 590 nm on a spectrophotometer and the data used to normalize Seahorse data or plotted directly to determine relative cell numbers.

Following a 24 h infection, cell viability was determined using a PI based cell exclusion assay as previously described (28). Cells were concomitantly stained with 5 μg/mL PI (Sigma-Aldrich), 20 μg/mL Hoechst 33342 (Sigma-Aldrich) and 50 μg/mL Hoechst 33258 (Sigma-Aldrich) for 30 min at room temperature. Total cell numbers were detected via Hoechst staining of nuclei (Blue channel: Ex 390 nm/Em 430 nm), and dying/dead cells were identified via positivity for PI staining (Orange channel: Ex 544 nm/EM 588 nm), using the Cytell Cell Imaging System and Cell Viability BioApp (GE Healthcare). 5 fields of view were acquired per treatment per well.

### Determination of mRNA Transcript and Protein Levels

RNA extractions from hMDMs and hAMs were performed using an RNeasy Mini Kit (Qiagen) following manufacturer’s instructions. RNA content and quality was quantified and assayed, respectively, using a Nanodrop (Thermo Fisher Scientific) and RNA reverse transcribed using the RevertAid First Strand cDNA Synthesis Kit (VWR). Catalogued pre-designed gene primer probes for *IL1*β, *TNF*α, *IL10*, *NF*κ*B*, *IL12a*, *IL18*, *PFKFB3*, *GAPDH*, *PKM2*, *G6PD*, *RPIA*, *CPT1A*, *FASN*, *GLS*, *IDO1*, and *18S* were purchased and real-time RT-qPCR was performed using Taqman Universal Master Mix (Applied Biosystems) on a QuantStudio 5 RT-qPCR System (Applied Biosystems). Relative quantitative data was obtained and analyzed utilizing the 2^–Δ^ ^Δ^ ^*Ct*^ method as previously described (27). Secreted protein levels of IL1β, IL10 (BioLegend ELISA Max Deluxe kits) and TNFα (Invitrogen ready-set-go kit) present in cell supernatants were quantified by ELISA, according to the manufacturer’s instructions.

### Characterizing the Effect of DFX on Real-Time Metabolism Profiles Utilizing the Seahorse XF_*e*_24 Analyzer

After 7–10 days of culturing and differentiating, hMDMs were scraped, counted and re-seeded at 2 × 10^5^ cells per well in a 24-well cell culture XF microplate (Seahorse Biosciences), incubated for 24 h, washed with complete RPMI, infected with Mtb and treated with DFX as described above. 24 h later, the cells were rinsed with assay medium [unbuffered DMEM supplemented with 10 mM glucose and 2 mM L-glutamine, pH 7.4 (Sigma-Aldrich)] before incubation with assay medium for 1 h at 37°C in a non-CO_2_ incubator. OCR and ECAR, reflecting oxidative phosphorylation and glycolysis, respectively, were measured before and after treatment with oligomycin (1 μM), FCCP (1 μM) and antimycin-A/rotenone (0.5 μM) (XF Cell Mito Stress Kit, Biosciences) using the Seahorse XF_*e*_24 analyzer (Seahorse Biosciences). Three baseline OCR and ECAR measurements were obtained over 20 min prior to injection of oligomycin, FCCP, and antimycin-A/rotentone. Three subsequent OCR and ECAR measurements were also obtained over 15 min following injection with oligomycin, FCCP and antimycin-A/rotenone. Oligomycin-induced glycolytic capacity was calculated by plotting ECAR as a percentage of baseline ECAR post oligomycin injection. ATP production was calculated by subtracting the OCR post oligomycin injection from baseline OCR prior to oligomycin addition and expressing residual OCR as a percentage of baseline OCR. Maximal respiratory capacity was calculated by plotting the percentage change in OCR post FCCP injection versus baseline OCR. Proton leak was calculated by subtracting percentage OCR versus baseline post antimycin-A/rotenone addition from percentage ATP production. Non-mitochondrial respiration was calculated by expressing residual OCR post antimycin-A/rotenone injection as a percentage of baseline OCR. The experiment was repeated a minimum of 5 times (*n* = 5–7), with at least two technical replicates. All measurements were normalized to cell number using the crystal violet assay.

### Characterizing the Effect of DFX on HIF1α Protein Levels Through Western Blotting Analyses

Upon completion of the infection, hMDMs were washed with pre-warmed PBS, the wash discarded and cells were scraped into 2 mL of fresh sterile pre-warmed PBS. The cells were centrifuged at 300 *g* for 5 min and the supernatant removed. The pelleted cells were lysed in radio-immunoprecipitation assay (RIPA) lysis buffer [50 mM Tris–HCL (pH 8), 1% v/v Triton X-100, 0.5% w/v sodium deoxycholate, 0.1% w/v sodium dodecyl sulfate, 150 mM NaCl, with the addition of protease (Thermo Fisher Scientific) and phosphatase inhibitor tablets (Sigma-Aldrich)]. Lysis was carried out at 4°C for 30 min. Lysates were centrifuged at 16,000 *g* for 10 min at 4°C and the supernatants removed to a fresh tube. The protein content of the supernatant was determined by BCA assay (Pierce). Cell lysates were boiled with equal volumes of SDS sample buffer [10 mM Tris–HCl (pH 6.8), 20% Glycerol (v/v), 4% sodium dodecyl sulfate (w/v), 0.001% bromophenol blue (w/v) containing 143 mM dithiothreitol] for 5 min. Equal amounts of lysates were resolved by sodium dodecyl sulfate polyacrylamide gel electrophoresis (SDS-PAGE). The transfer of separated proteins to PVDF membrane was performed by wet blotting. The PVDF membranes were blocked with blocking buffer containing 5% (w/v) dried skimmed milk in Tris-Buffered saline Tween (TBST) (0.1% (v/v) Tween-20 in TBS) at room temperature for 1 h. Following blocking, the membrane was incubated with purified mouse anti-human HIF1α overnight at 4°C (in TBST) (BD Biosciences), followed by incubation with secondary goat anti-mouse IgG peroxidase conjugated antibody (in TBST) (Millipore) for 1 h at room temperature. The immunoblots were developed using enhanced chemiluminescence (ECL) (MyBio) and visualized using a chemiluminescence imaging system (Fusion FX).

### Compounds Used to Determine the Functional Mechanism of DFX in Mtb-Infected hMDMs

hMDMs infected with Mtb were treated with 5 mM 2-deoxy-d-glucose (2DG) (Sigma-Aldrich) 1 h prior to infection. Uninfected and Mtb-infected hMDMs were treated with 25 μM PX-478 or with 1 mM of cell permeable α-KG derivative 1 h prior to infection, as previously described (29–31).

### Quantifying HIF1α-IL1β Promoter Binding Enrichment in Mtb-Infected hMDMs Treated With DFX Through Chromatin Immunoprecipitation Coupled With Quantitative PCR (ChIP-qPCR)

PBMCs were cultivated and differentiated into hMDMs in 6 well plates, stimulated with Mtb iH37Rv and treated with DFX as described above. The cell monolayer was washed twice with PBS and harvested by cell scraping. Cells were transferred to 15 mL falcon tubes containing 1% formaldehyde solution (Sigma-Aldrich). Cells were crosslinked for 10 min at room temperature before the reaction was quenched with 0.125 M glycine (Sigma-Aldrich). Cross-linked cells were washed twice with PBS and lysed in 5 mL SDS lysis buffer (100 mM NaCl, 50 mM Tris-Cl, 5 mM EDTA, 0.02% NaN3, and 1% SDS) containing protease inhibitors (1 μg/mL leupeptin, 1 μg/mL aprotenin, 10 μM PMSF; Sigma-Aldrich). Cell lysates were re-suspended in ice-cold nuclear lysis immunoprecipitation (IP) buffer containing two volumes SDS lysis buffer and 1 volume dilution buffer (100 mM NaCl, 50 mM Tris-Cl, 5 mM EDTA, 0.02% NaN3, 5% Triton X-100; Sigma-Aldrich). Cell lysates were sonicated to acquire chromatin fragments ranging from 100–300 bp. The sonicator (130 watt ultrasonic sonicator, Sonics) was set to 62% power with alternating time intervals of 30 s “ON” and 30 s “OFF” for a total of 20 min “ON” time. To verify optimum DNA size fragmentation, phenol/chloroform DNA extraction and ethanol-precipitation was performed (see section “Phenol-Chloroform Extraction and Ethanol Precipitation”), and purified DNA was run on a 1% agarose gel (Sigma-Aldrich). 50 ug/mL of cell lysate was transferred into 1.5 mL tubes (Eppendorf) and centrifuged at 14,000 *g* for 30 min. The supernatant was collected in 1.5 mL safe lock tubes (Eppendorf) and immunoprecipitated overnight at 4^*o*^C with 2 ug/mL anti-HIF-1α IgG (Abcam) or 1 ug/mL IgG control (Sigma-Aldrich). Immune complexes were recovered by adding 70 μL Tris-Cl Dynabeads (Invitrogen) and incubated on a rotator for 4 h at room temperature. Beads were washed sequentially with 1 mL of the following buffers for 5 min at 4°C: Mixed Micelle Buffer (3 times), Buffer 500 (2 times) and LiCl Detergent Wash Buffer (2 times). The last wash was performed in TE buffer for 2 min at 4°C. To elute, 250 μL of ChIP elution buffer was added to the beads and incubated at 65°C for 1 h. The beads were pelleted and the supernatant was incubated overnight at 65°C to reverse the crosslinking. The eluate was phenol/chloroform-extracted and ethanol-precipitated. DNA was resuspended in 200 μL of water for standard real-time qPCR, utilizing SensiFAST^TM^ SYBR Hi-Rox mix and forward and reverse probes for *IL1*β (see the resources table for sequences). qPCR was run at 95°C for 10 min (1.6°C/s), followed by 40 cycles of 95°C for 15 s (1.6°C/s) and 61°C for 30 s (1.6°C/s) and analyzed in conjunction with melt curve analysis.

### Phenol-Chloroform Extraction and Ethanol Precipitation

Sonicated DNA was treated with 1 μg/mL RNase (Thermo Fisher Scientific) and 1 μL/mL phosphatase K (New England Biolabs) for 20 min at 37^*o*^C and 30 min at 55^*o*^C, respectively. DNA was diluted in TE buffer and 1 volume phenol/chloroform (Invitrogen) was added. Samples were vortexed vigorously and centrifuged for 10 min at 14,000 *g*. The upper aqueous DNA phase was transferred to a new Eppendorf tube and 1mL of ethanol containing 40 μL 3M sodium acetate (pH 5.5) and 2 μL glycogen (Sigma-Aldrich) was added. DNA was precipitated at −80^*o*^C for 30 min and pelleted by centrifugation at 14,000 *g* for 20 min. Contaminants were removed with 70% ethanol and the DNA pellet was re-suspended in dH2O.

### Examining the Antibacterial Effect of DFX on Colony Forming Units of Mtb

A total of 72 h post infection with Mtb, in the presence and absence of DFX, the supernatant was removed from each well and centrifuged at 3000 *g* for 10 min. The supernatant was removed and the pellet kept. 0.5 mL PBS lysis buffer (supplemented with 0.1% Triton-X 100) was added to each well and incubated for 5 min at room temperature to lyse the infected hMDMs. The hMDMs were subsequently scraped and added to the corresponding pellet. 0.5 mL PBS was used to wash each well which was also added to the equivalent pellet. The contents from each well were then homogenized with a 25 gauge needle, using a 1 mL syringe, to re-suspend the Mtb. For the CFUs assay, three dilutions (10^–1^, 10^–2^, and 10^–3^) of each suspension were spread, in triplicate, on Middlebrook agar 7H10 (Becton Dickinson) supplemented with oleate-ADC (Becton Dickinson). The plates were sealed and incubated at 37°C. Over the following 21 days post-treatment, numbers of CFUs visible on each plate were recorded allowing viability of Mtb to be calculated for each treatment.

### Statistical Analysis

Data was analyzed using Graph Pad Prism 5 software (Graph Pad Prism, San Diego, CA, United States). A Friedman ANOVA test with a Bonferroni *post hoc* test was used to investigate the effect of 2DG on DFX-induced IL1β levels in hMDMs. Real-time qPCR data was normalized using the 2^–ΔΔ*Ct*^ method and statistically analyzed using Friedman ANOVA with Bonferroni *post hoc* tests. ELISA and real-time Seahorse extracellular metabolic flux analyses were statistically analyzed using Friedman ANOVA with Bonferroni *post hoc* tests. A Wilcoxon signed rank test was used to test for a statistical difference in glycolytic capacity between uninfected and Mtb-infected hMDMs. Hypoxia and mechanistic analyses were statistically analyzed using Friedman ANOVA with Bonferroni *post hoc* tests. One sample *t*-tests were used to examine fold change differences in cytokine secretions (normoxia analyses). A Friedman ANOVA with Dunn’s multiple comparison tests were utilized to test for statistical differences in the effect of DFX on Mtb uptake and cell viability. Differences of *P* < 0.05 (^∗^), *P* < 0.01 (^∗∗^), and *P* < 0.001 (^∗∗∗^) were considered statistically significant.

## Results

### Timing Optimization of DFX Treatment

DFX is a clinically approved iron chelator which is effective for long-term iron chelation therapy (32). To explore if DFX demonstrates potential as a HDT during early bacterial infection, we examined if DFX could enhance macrophage metabolism and function in a human macrophage model of Mtb infection. To achieve this, we first determined the optimal time to treat hMDMs with DFX, relative to the time that the hMDMs were infected with Mtb. We infected hMDMs, from healthy blood donors, with Mtb H37Ra for 3 h, washed off any residual non-phagocytosed Mtb, and treated with DFX 24 h prior to infection, at the time of infection or 3 h post infection. As IL1β and TNFα are known to be crucial cytokines in the early host response to Mtb infection (33, 34), secreted levels of these cytokines were assayed to assess the effect of DFX on macrophage function across the three time points. In uninfected hMDMs, DFX treatment had no effect on IL1β or TNFα secretion (Supplementary Figures S1A,B). In Mtb-infected hMDMs, however, DFX boosted secreted levels of IL1β and TNFα, exhibiting significant effects on IL1β at 0 and 3 h post infection (Supplementary Figure S1A), while displaying a significant effect on TNFα levels when added 24 h before infection (Supplementary Figure S1B). To ensure DFX had no effect on macrophage uptake of Mtb, which could subsequently affect the levels of these cytokines, the phagocytic ability of DFX was subsequently examined to rule differences in Mtb uptake. DFX exhibited no significant effect on uptake (Supplementary Figure S1C). As DFX induced the highest levels of IL1β 3 h post infection, we selected this time point for subsequent experimental analyses. Moreover, using a propidium iodide (PI)-based cell exclusion assay, we found that DFX did not affect cell viability in hMDMs stimulated with iH37Rv at this time point (Supplementary Figure S1D).

### DFX Enhances the Transcription of Glycolytic Metabolism Genes in hMDMs Infected With Mtb

**FIGURE 1:**
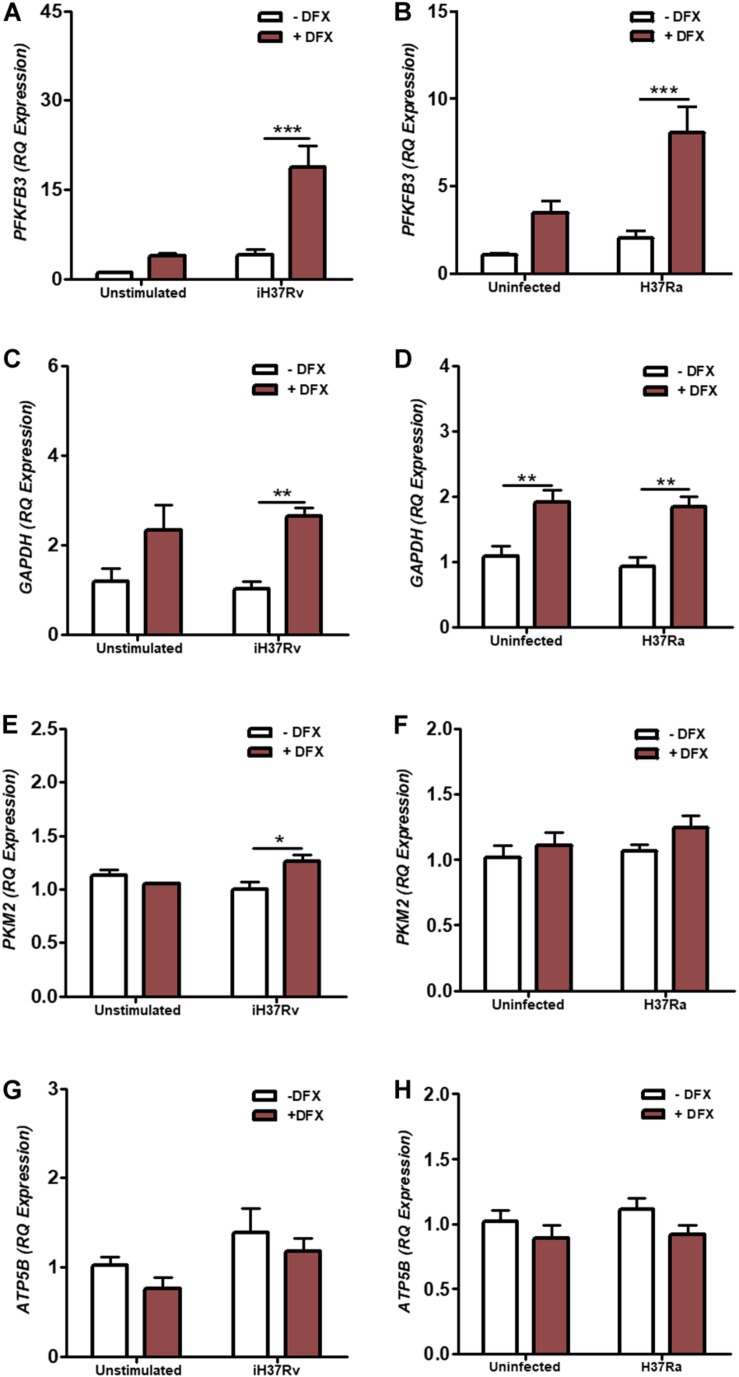
DFX enhances the expression of glycolytic metabolism genes in hMDMs infected with Mtb. hMDMs, differentiated from PBMCs isolated from healthy blood donors, were stimulated with Mtb iH37Rv or infected with Mtb H37Ra for 3 h, washed to remove unphagocytosed Mtb, and were treated with DFX (100 μM). 24 h post infection transcript levels of **(A,B)** *PFKFB3* (*n* = 5), **(C,D)** *GAPDH* (*n* = 5) **(E,F)**, *PKM2* (*n* = 3–5) (representing glycolysis), and **(G,H)** *ATP5B* (*n* = 5) (representing oxidative phosphorylation) were determined by RT-qPCR. Bars denote mean ± SEM. *P < 0.05, **P < 0.01, and ***P < 0.001 (Two-way ANOVA with Bonferroni *post hoc* tests).

*In vitro* studies in hMDMs and hAMs, and *in vivo* studies in mice, support the idea of a metabolic reprogramming toward glycolysis upon Mtb infection (8–10). Moreover, this shift toward glycolysis during Mtb infection is coupled to the ability of human macrophages to produce mature IL1β and diminish Mtb burden, as bactericidal activity and IL1β levels are reduced when glycolysis is abrogated in macrophages (10). The transcription factor HIF1α, known to regulate various cellular processes including glycolytic metabolism, has previously been shown to be stabilized by DFX in various cell types (35–37). As glycolytic metabolism is integral to host defense during early Mtb infection, we investigated the effect of DFX on glycolytic metabolism in hMDMs infected with Mtb. To examine this, transcript levels of *PFKFB3*, *GAPDH*, and *PKM2* were determined, as these genes encode proteins at the beginning, middle and end stages of the glycolytic pathway, respectively. We found that DFX-treated hMDMs, stimulated with Mtb iH37Rv (Figures 1A,C,E) and infected with Mtb H37Ra (Figures 1B,D,F), exhibited enhanced transcript levels of *PFKFB3* (Figures 1A,B), *GAPDH* (Figures 1C,D) and *PKM2* (Figure 1E). Furthermore, DFX increased transcript levels of *GAPDH* in unstimulated/uninfected hMDMs (Figure S1D and Supplementary Figure S2B). DFX did not affect *ATP5B* transcript levels, a gene marker of OXPHOS, in unstimulated/uninfected, Mtb iH37Rv-stimulated and Mtb H37Ra*-*infected hMDMs (Figures 1G,H). DFX also significantly increased these glycolytic genes in lipopolysaccharide (LPS)-stimulated hMDMs (Supplementary Figures S2A–C), without affecting *ATP5B* levels in the process (Supplementary Figure S2D).

We also wished to gain insight into how DFX potentially alters the activity of alternative metabolic pathways. *G6PD* and *RPIA* transcript levels (representing the oxidative and non-oxidative pathways of the pentose phosphate pathway, respectively), *CPT1A* and *FASN* transcript levels (representing fatty acid oxidation and fatty acid synthesis, respectively) and *GLS* and *IDO1* transcript levels (representing glutamine and tryptophan metabolism, respectively) were similarly assessed in Mtb iH37Rv-stimulated and Mtb H37Ra*-*infected hMDMs (Supplementary Figure S3). While not altering *G6PD* transcript levels in Mtb iH37Rv*-*stimulated hMDMs (Supplementary Figure S3A), DFX significantly reduced *RPIA* transcript levels in Mtb iH37Rv-stimulated hMDMs (Supplementary Figure S3B). DFX significantly reduced levels of the *CPT1A* transcript (Supplementary Figure S3C), but had no significant effect on the other fatty acid metabolism transcript, *FASN*, in hMDMs stimulated with Mtb iH37Rv (Supplementary Figure S3D). Moreover, DFX significantly enhanced levels of the *GLS* transcript in iH37Rv-stimulated hMDMs (Supplementary Figure S3E), without altering *IDO1* levels in hMDMs stimulated with iH37Rv (Supplementary Figure S3F). Interestingly, DFX did not significantly alter the expression of these metabolic genes in uninfected hMDMs or in hMDMs infected with the live Mtb H37Ra strain. Moreover, DFX significantly reduced *CPT1A* in unstimulated and LPS-stimulated hMDMs, without affecting transcript levels of *G6PD*, *RPIA*, *FASN*, *GLS*, or *IDO1* (Supplementary Figure S4). Thus, the increase in the glycolytic genes could be indicative that DFX induces a shift to glycolytic metabolism in Mtb-infected human macrophages, however, further metabolic analyses were required to confirm this observation.

### DFX Induces the Warburg Effect in Unstimulated and Mtb-Stimulated hMDMs

**FIGURE 2:**
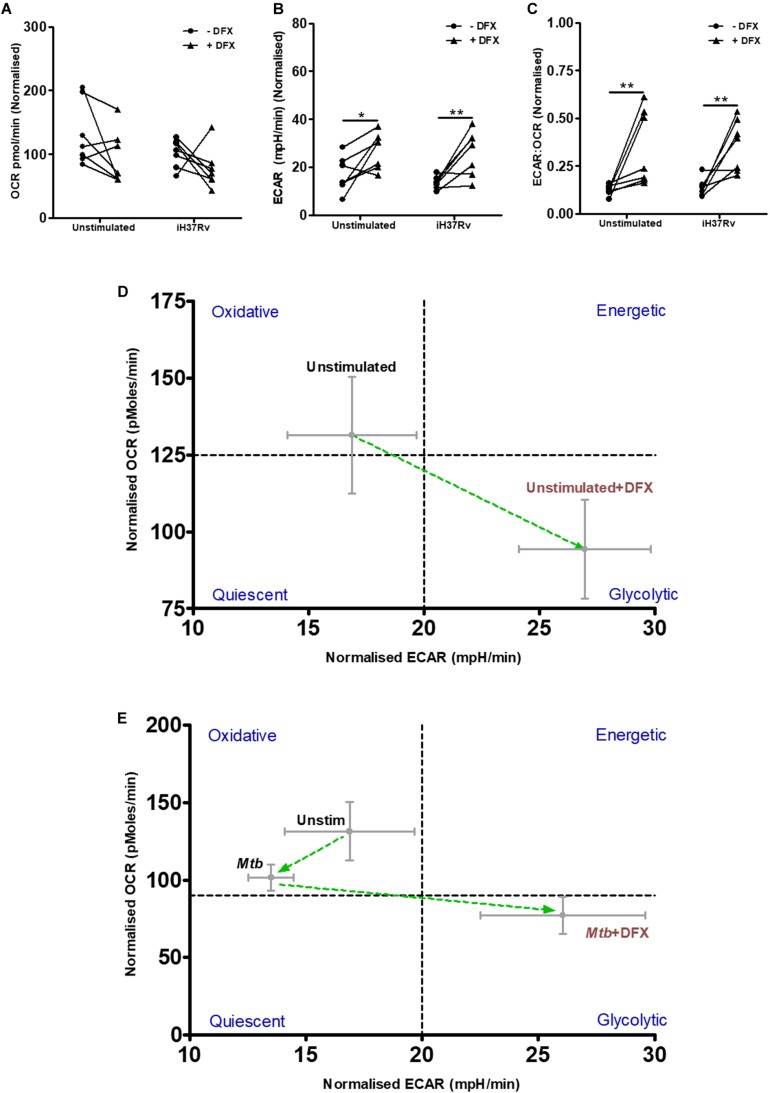
DFX induces the Warburg effect in unstimulated and Mtb-infected hMDMs. The effect of DFX on real-time baseline **(A)** OCR and **(B)** ECAR profiles, representing oxidative phosphorylation and glycolysis, respectively, was determined utilizing Seahorse extracellular flux assays in uninfected and Mtb iH37Rv-stimulated hMDMs 24 h post infection, in the absence and presence of DFX (*n* = 7). **(C)** The ECAR:OCR ratio was generated to measure the reliance of one metabolic pathway over another (*n* = 7). The immunometabolic shift from DFX treatment is illustrated by the metabolic phenograms in **(D)** uninfected and **(E)** Mtb-infected hMDMs. *P < 0.05 and **P < 0.01 (Two-way ANOVA with Bonferroni *post hoc* tests).

To validate our findings at the transcript level that DFX alters macrophage metabolism, the effect of DFX on glycolysis and OXPHOS was determined in real-time, utilizing Seahorse extracellular flux assays. To do this, extracellular acidification rate (ECAR), which is an indication for the release of protons produced during glycolysis, and oxygen consumption rate (OCR), which indicates cellular respiration and energy production through OXPHOS, was determined in unstimulated hMDMs and Mtb-stimulated hMDMs treated with or without DFX (Figure 2). Mtb-stimulated hMDMs treated with DFX demonstrated no difference in OCR levels (Figure 2A). Mtb-stimulated hMDMs treated with DFX, however, exhibited significantly enhanced ECAR levels (Figure 2B). The ECAR:OCR ratio, which can be utilized to determine a cell’s preference for one pathway over another and is a measure of the relative magnitude of glycolysis versus OXPHOS, showed that Mtb-stimulated hMDMs treated with DFX exhibited a preference for glycolysis (Figure 2C). Interestingly, the effect of DFX was not restricted to Mtb-stimulated hMDMs, as unstimulated hMDMs treated with DFX also exhibited significantly increased ECAR (Figure 2B) and significantly elevated ECAR:OCR ratios (Figure 2C). This metabolic shift from oxidative metabolism to glycolytic metabolism upon DFX treatment, is depicted by the phenograms in Figures 2D,E, respectively. DFX significantly increased ECAR (Supplementary Figure S2E), reduced OCR (Supplementary Figure S2F) and increased ECAR:OCR ratios (Supplementary Figure S2G) in LPS-stimulated hMDMs. This DFX-induced glycolytic metabolism can be illustrated by the metabolic phenogram (Supplementary Figure S2H).

### DFX Boosts Glycolytic Capacity in Unstimulated and Mtb-Stimulated hMDMs

**FIGURE 3:**
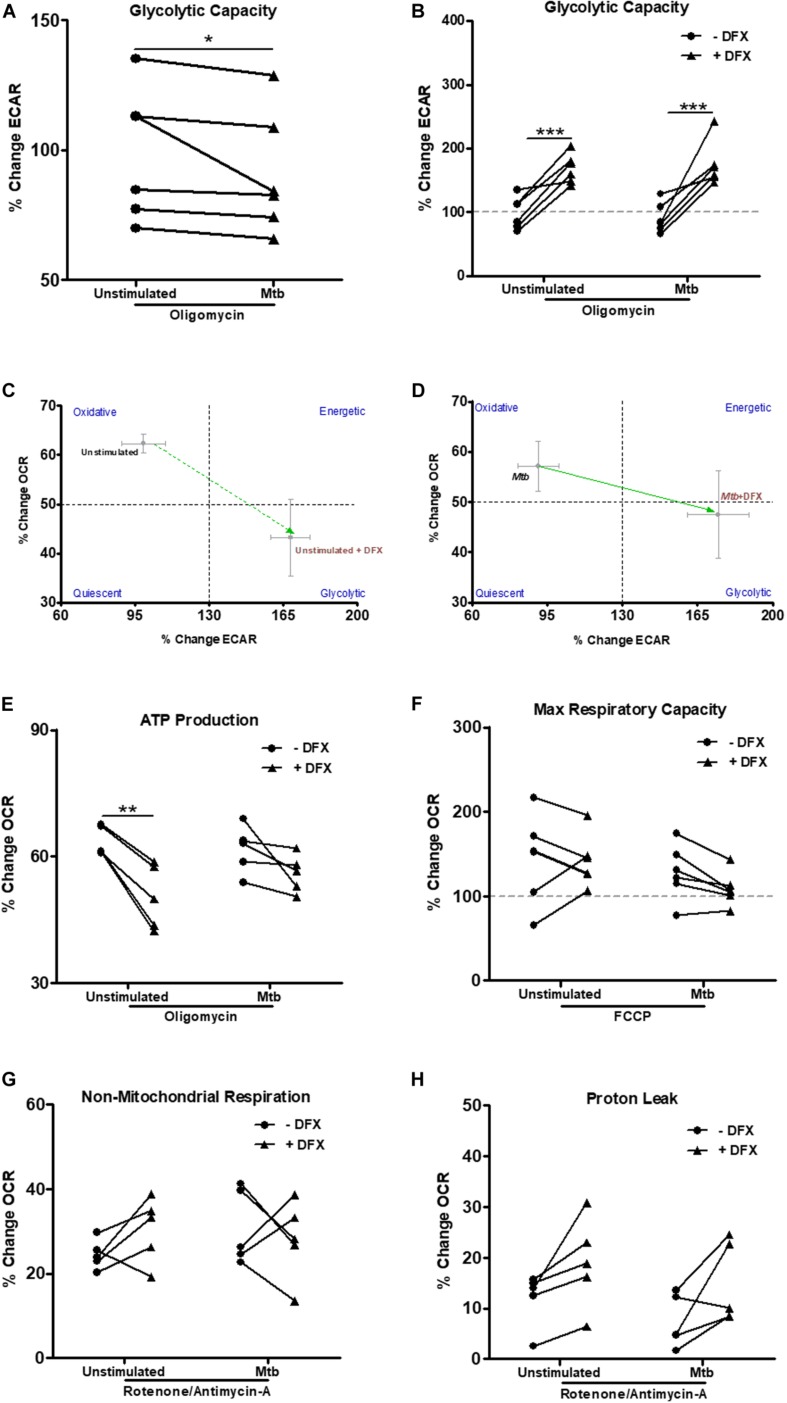
DFX restores glycolytic capacity in uninfected and Mtb-stimulated hMDMs. Injection of specific mitochondrial function inhibitors allowed additional metabolic parameters to be assessed utilizing Seahorse extracellular flux assays in uninfected and Mtb iH37Rv-stimulated hMDMs 24 h post infection, in the absence and presence of DFX (*n* = 6). **(A)** After treating hMDMs with the ATP synthase inhibitor oligomycin (1 μM), glycolytic capacity, as measured by ECAR, was evaluated (*n* = 6). **(B)** The ability of DFX to restore this glycolytic capacity was subsequently examined in these cells (*n* = 6). The resulting DFX-mediated immunometabolic shifts in glycolytic capacity in **(C)** unstimulated and **(D)** Mtb iH37Rv-stimulated hMDMs can be illustrated by metabolic phenograms (*n* = 6). **(E)** In parallel with oligomycin treatment, the effect of DFX on ATP production, as measured by OCR, was also determined (*n* = 6). **(F)** DFX-induced alterations in maximal respiratory capacity was also evaluated upon FCCP treatment (FCCP) (1 μM) (*n* = 6). Following rotenone/antimycin-A treatment (0.5 μM), the effect of DFX treatment on **(G)** non-mitochondrial respiration and **(H)** proton leak was determined (*n* = 5). *P < 0.05, **P < 0.01, and ***P < 0.001 (**A:** Wilcoxon signed rank test; **B–H**: two-way ANOVA with Bonferroni *post hoc* tests). Dotted lines (at 100%) correspond to baseline conditions in graphs **(B,F)**.

The ability of Mtb to cause significant mitochondrial inner membrane disruption in macrophages leading to increased Mtb survival, growth and successful infection has been well documented in recent decades (38, 39). Accordingly, we examined if DFX could support macrophage function during periods of mitochondrial instability and dysfunction. To do this, various metabolic parameters were assessed in DFX-treated unstimulated and Mtb-stimulated hMDMs, namely glycolytic capacity, ATP production, maximal respiratory capacity, non-mitochondrial respiration and proton leak (Figure 3). This was achieved utilizing Seahorse extracellular flux assays, by injecting specific inhibitors of mitochondrial function and assessing their effect on ECAR and OCR rates over a 15 min period. After treating hMDMs with the ATP synthase inhibitor, oligomycin, the capacity of hMDMs to increase glycolysis to compensate for the lack of ATP being produced through OXPHOS, was evaluated in unstimulated and Mtb-stimulated hMDMs. After oligomycin injection, Mtb-stimulated cells exhibited significantly reduced ability to increase their glycolytic capacity compared to unstimulated hMDMs, as indicated by the reduced percentage change in ECAR versus baseline ECAR (Figure 3A). We found, however, that DFX significantly increased the ability of both unstimulated and Mtb-stimulated hMDMs to increase their glycolytic capacity after treatment with oligomycin (Figure 3B). Interestingly, DFX could not restore this glycolytic capacity in hMDMs stimulated with LPS (Supplementary Figure S2I). These DFX-mediated immunometabolic shifts in glycolytic capacity are depicted by the metabolic phenograms in Figures 3C,D. These data demonstrate that DFX exhibits the ability to restore glycolytic flux in Mtb-stimulated hMDMs during periods of mitochondrial dysfunction.

Iron metabolism is a fundamental component in the synthesis of heme and iron-sulfur cluster-containing proteins, which have crucial roles in the operation of the electron transport chain (40, 41). Therefore, we also investigated the effect of DFX on parameters linked to mitochondrial oxidative metabolism. Treatment with oligomycin allows the degree of ATP production attributed to ATP synthase activity to be determined, as measured by the percentage change in OCR upon oligomycin treatment versus baseline OCR. Unstimulated hMDMs displayed reduced dependence on using oxygen for the production of ATP when treated with DFX, however, this was not observed in Mtb-stimulated hMDMs (Figure 3E). After uncoupling the mitochondrial membrane with trifluorocarbonylcyanide phenylhydrazone (FCCP), which allows the maximal respiratory capacity to be determined, we found no difference in unstimulated and Mtb-stimulated hMDMs upon DFX treatment (Figure 3F). Furthermore, following combined rotenone-antimycin-A treatment, DFX had no significant effect on non-mitochondrial respiration (Figure 3G) or proton leak (Figure 3H) in unstimulated or Mtb-stimulated hMDMs, as measured by the percentage change in OCR versus baseline after injection with rotenone-antimycin-A. Furthermore, DFX did not alter ATP production, maximal respiratory capacity, non-mitochondrial respiration or proton leak in hMDMs stimulated with LPS (data not shown).

### DFX Enhances IL1β and TNFα Levels in Human Macrophages Infected With Mtb

**FIGURE 4:**
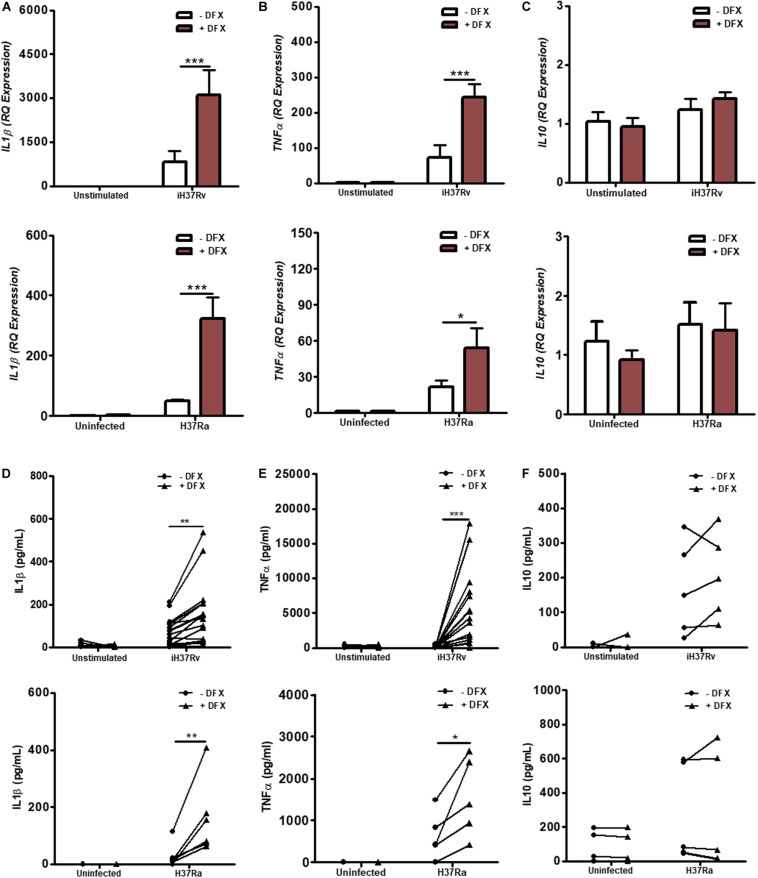
DFX supports immune function by enhancing transcript and protein levels of IL1β and TNFα in hMDMs infected with Mtb. hMDMs, differentiated from PBMCs isolated from healthy blood donors, were stimulated with Mtb iH37Rv or infected with Mtb H37Ra for 3 h, washed to remove unphagocytosed Mtb, and were treated with DFX (100 μM). 24 h post infection transcript (**A–C**; *n* = 4–5) and protein (**D–F**; *n* = 5–17) levels of IL1β **(A,D)**, TNFα **(B,E)** and IL10 **(C,F)** were quantified by RT-qPCR and ELISA. Bars denote mean ± SEM. *P < 0.05, **P < 0.01, and ***P < 0.001 (Two-way ANOVA with Bonferroni *post hoc* tests).

The intrinsic relationship between iron and the generation of a pro-inflammatory response also prompted us to further explore the possibility that DFX could be utilized to boost the macrophage response during early infection (42). As IL1β and TNFα are known to be fundamental during the early host response to Mtb infection (12, 13), these cytokines were assayed to assess the effect of DFX on macrophage function. Using healthy blood donors, we stimulated and infected hMDMs, respectively, with Mtb iH37Rv or Mtb H37Ra for 3 h, washed off any residual non-phagocytosed Mtb, and subsequently treated with DFX. 24 h post infection, real-time RT-qPCR and ELISAs were employed to assess the effect of DFX on transcript and protein levels of IL1β and TNFα. Transcript and protein levels of the anti-inflammatory cytokine IL10 were also examined, the expression of which has been shown to promote tuberculosis disease progression and Mtb pathogen persistence in CBA/J mice (43), and blocks phagosome maturation in hMDMs and hAMs (44). We found that DFX treatment significantly boosted *IL1*β (Figure 4A) and *TNF*α (Figure 4B) transcript levels in hMDMs stimulated with Mtb iH37Rv and infected with live attenuated Mtb H37Ra, without affecting transcript levels of *IL10* (Figure 4C). When secreted levels of these cytokines were examined, we found that DFX significantly increased secreted levels of both IL1β (Figure 4D) and TNFα (Figure 4E) from hMDMs stimulated with Mtb iH37Rv and infected with Mtb H37Ra. DFX did not alter secreted levels of IL10 (Figure 4F). Moreover, DFX failed to alter transcript or protein levels of IL1β, TNFα or IL10 in unstimulated/uninfected hMDMs (Figures 4A–F). CCL5, CXCL8, CXCL10, NFκB, IL12a, and IL18 are additional cell mediators known to play supportive roles in immune defense against various pathogens including Mtb (45–49). We found that DFX boosted *CXCL8* (Supplementary Figure S5C) and *CXCL10* (Supplementary Figure S5E) transcript levels in hMDMs stimulated with Mtb iH37Rv and infected with live attenuated Mtb H37Ra, without altering transcript levels of *CCL5* (Supplementary Figure S5A). DFX also boosted secreted levels of CXCL8 (Supplementary Figure S5D), but did not significantly alter secreted levels of CCL5 (Supplementary Figure S5B) or CXCL10 (Supplementary Figure S5F). DFX failed to alter transcript or protein levels CCL5, CXCL8 or CXCL10 in unstimulated/uninfected hMDMs (Supplementary Figure S5). We also found that DFX significantly boosted *NF*κ*B* (Supplementary Figure S6A) and *IL12a* (Supplementary Figure S6B) transcript levels in hMDMs stimulated with Mtb iH37Rv, but did not have any effect in unstimulated hMDMs. DFX treatment did not affect *IL18* transcript levels in unstimulated and Mtb iH37Rv stimulated hMDMs (Supplementary Figure S6C). In hMDMs stimulated with LPS, DFX boosted transcript and proteins levels of IL1β (Supplementary Figures S7A,D), without altering levels of IL10 (Supplementary Figures S7C,F). DFX boosted transcript levels of TNFα (Supplementary Figure S7B), but did not alter corresponding protein levels (Supplementary Figure S7E). DFX boosted transcript and proteins levels of CXCL8 (Supplementary Figures S8B,E), but did not alter levels of CCL5 (Supplementary Figures S8A,D) or CXCL10 (Supplementary Figures S8C,F).

### DFX Boosts IL1β and Glycolytic Gene Expression in Human Alveolar Macrophages Infected With Virulent H37Rv Mtb

**FIGURE 5:**
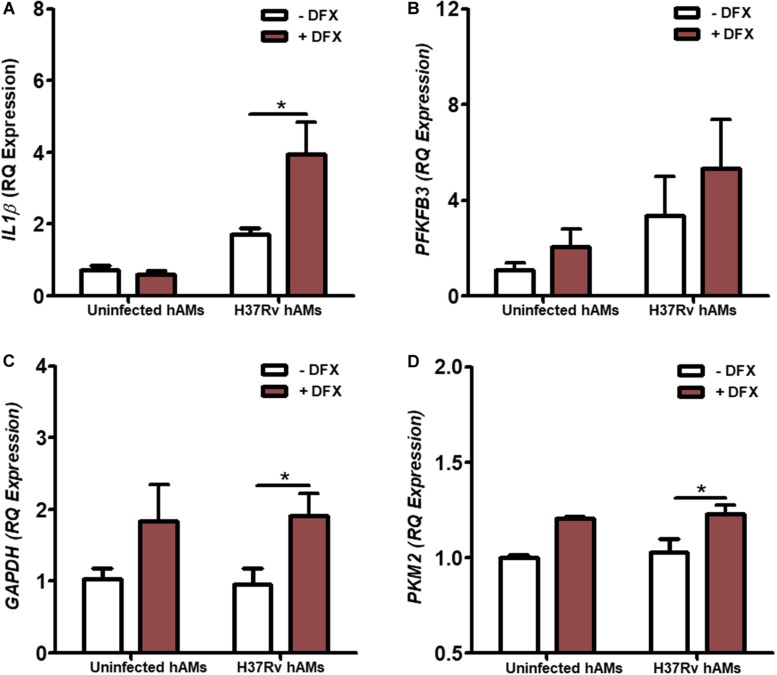
DFX enhances IL1β and glycolytic genes in primary human alveolar macrophages infected with Mtb. hAMs were adherence purified from BAL fluids obtained from consenting patients during bronchoscopy. hAMs were infected with virulent Mtb H37Rv and were treated with DFX (100 μM) 3 h post infection. 24 h post infection transcript levels of **(A)** *IL1*β, **(B)** *PFKFB3*, **(C)** *GAPDH* and **(D)** *PKM2* were examined in uninfected and Mtb H37Rv-infected hAMs, in the absence and presence of DFX (*n* = 3–4). Bars denote mean ± SEM. *P < 0.05 (Unpaired two-way ANOVA with Bonferroni *post hoc* tests).

hAMs are the first cells to be infected during Mtb infection *in vivo* (50, 51). To validate the clinical utility of DFX in supporting inflammatory and immunometabolic profiles during early Mtb infection, the effect of DFX on transcript levels of *IL1*β, *PFKFB3*, *GAPDH*, and *PKM2* was examined. To do this, hAMs were obtained at bronchoscopy after informed consent, and infected with virulent Mtb H37Rv for 24 h and treated with DFX 3 h post infection. We found that DFX significantly boosted *IL1*β transcript levels in Mtb H37Rv*-*infected hAMs (Figure 5A). Moreover, despite failing to affect *PFKFB3* transcript levels (Figure 5B), DFX significantly enhanced both *GAPDH* (Figure 5C) and *PKM2* (Figure 5D) transcript levels in Mtb H37Rv-infected hAMs.

### DFX Primarily Modulates Cell Function in Mtb-Infected Human Macrophages by Stabilizing HIF1α

**FIGURE 6:**
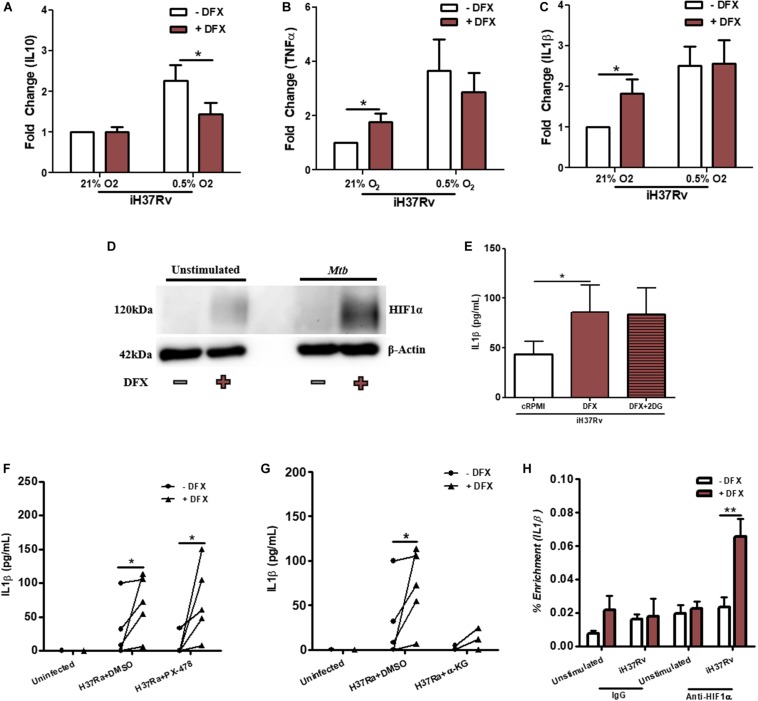
DFX modulates Mtb-infected human macrophages by stabilizing HIF1α. The effect of DFX treatment on secreted levels of **(A)** IL10, **(B)** TNFα, and **(C)** IL1β were quantified in unstimulated and Mtb iH37Rv-stimulated hMDMs under normoxia (21% O_2_) or hypoxia (0.5% O_2_) (*n* = 10–13). **(D)** Western blotting was subsequently performed to examine HIF1α protein levels in the presence and absence of DFX in unstimulated hMDMs and hMDMs stimulated with Mtb iH37Rv (blot representative of *n* = 3). **(E)** The glycolytic inhibitor 2DG (5 mM) was used to block glycolytic metabolism in Mtb iH37Rv-stimulated hMDMs. Supernatants were harvested and IL1β was quantified 24 h post infection (*n* = 8). **(F)** The early HIF1α transcription/translation inhibitor PX-478 (25 μM) and **(G)** blockade of downstream HIF1α-IL1β promoter binding with TFMB α-KG (1mM) was employed to assess the role of HIF1α in DFX-induced IL1β in Mtb H37Ra-infected hMDMs (*n* = 5; DMSO concentration: 0.2%). **(H)** ChIP-qPCR was used to determine if DFX treatment could enhance HIF1α-IL1β promotor binding in unstimulated and Mtb iH37Rv-stimulated hMDMs (*n* = 3). Bars denote mean ± SEM. *P < 0.05 and **P < 0.01 (**A–C,F–H**: Two-way ANOVA with Bonferroni *post hoc* tests; **E:** Friedman test with Dunn’s multiple comparison test).

The transcription factor HIF1α is known to be stabilized by DFX in various cell types (35–37). DFX, through the stabilization of HIF1α, is also known to induce the secretion of IL1β and regulate glycolytic metabolism (30). Indeed, the addition of iron ammonium sulfate blocks the DFX-mediated induction of HIF1α in Hep3B cells and supplementation with iron chloride has been shown to abrogate DFX-mediated release of IL1β from LPS-stimulated hAMs (37, 52). Based on these observations, we hypothesized that DFX may be primarily mediating its inflammatory and immunometabolic effects in Mtb-infected macrophages through HIF1α. To test this, we first examined whether hypoxic conditions, which stabilize HIF1α, could mimic the effect of DFX, as this may indicate that DFX exerts its functional effect through HIF1α stabilization. Under 0.5% oxygen (i.e., hypoxia), DFX treated Mtb-stimulated hMDMs exhibited significantly reduced levels of IL10 compared to normoxic conditions (Figure 6A), however, DFX failed to alter TNFα (Figure 6B) or IL1β (Figure 6C) levels when under hypoxic conditions. DFX’s failure to further increase IL1β secretion under hypoxia, when HIF1α was already stabilized, strengthened our hypothesis that HIF1α plays a role in mediating the effect of DFX. To interrogate this further, western blot analyses were undertaken to characterize expression levels of HIF1α protein under normoxic conditions. HIF1α expression was increased upon treatment with DFX in Mtb-stimulated hMDMs (Figure 6D and Supplementary Figure S9A). Furthermore, Mtb-stimulated hMDMs treated with the glycolytic inhibitor 2DG failed to block DFX-induced increases in IL1β, indicating that DFX-induced increases in IL1β are likely to be independent of the observed alterations on glycolysis (Figure 6E). Interestingly, PX-478, previously used to target *de novo* HIF1α production in neoplastic tissues (31), failed to block DFX-induced increases in IL1β (Figure 6F). PX-478 also exhibited no significant effect on TNFα levels in the same cells (Supplementary Figure S9B). However, selectively abrogating the interaction between HIF1α and IL1β by blocking downstream HIF1α-IL1β promoter binding with an α-KG analog derivative, previously described to block HIF1α-IL1β promoter binding in LPS-stimulated murine macrophages (30), blocked DFX-induced increases in IL1β protein levels in Mtb*-*infected hMDMs (Figure 6G). α-KG exhibited no statistically significant effect on TNFα levels in the same cells (Supplementary Figure S9C). Additionally, ChIP-qPCR analyses showed that DFX treatment significantly enhanced HIF1α-*IL1*β promoter binding in Mtb*-*stimulated hMDMs after 24 h, as reflected by enhanced HIF1α-mediated enrichment of *IL1*β transcript levels (Figure 6H). This enrichment of HIF1α-*IL1*β promoter binding could not be observed after 8 h (Supplementary Figure S9D). Therefore, these data show that DFX partly modulates its effect in human macrophages through the stabilization of HIF1α.

In addition to DFX’s ability to boost immune function and enhance glycolytic function, we also examined if DFX exhibited bactericidal potential. DFX did not significantly alter colony formation units (CFUs) in hMDMs infected with Mtb H37Ra (Supplementary Figures S9E,F). Thus these analyses provide some insight into the use of DFX as a possible adjunct HDT option during early Mtb infection, and provides insight into its ability to enhance inflammatory and immunometabolic profiles for other infectious diseases.

## Discussion

**FIGURE 7:**
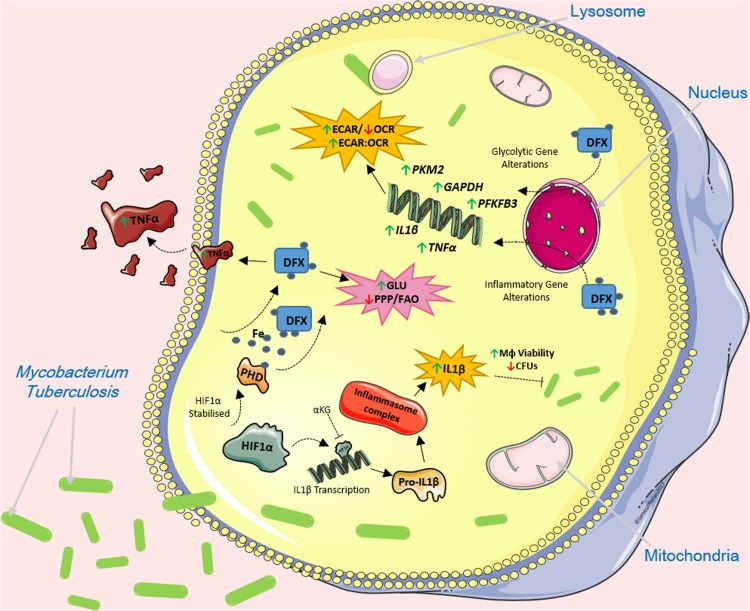
The effect of DFX on Mtb-infected macrophages. DFX supports immune function by enhancing transcript and protein levels of IL1β and TNFα in hMDMs infected with Mtb. DFX enhanced glycolytic metabolism in hAMs and hMDMs infected with Mtb. Moreover, DFX differentially affects alternative metabolic pathways, decreasing pentose phosphate pathway activity and fatty acid oxidation. DFX modulates its effect in Mtb-infected human macrophages through the stabilization of HIF1α. Image produced with the aid of Servier Medical Art software (see copyright license at https://smart.servier.com).

Recent insights into pathogen-host interactions and early host innate immune responses are leading to the identification and development of a wide range of HDTs. As a result, the re-purposing of current FDA-approved drugs as HDTs is now becoming a viable adjunct to standard antimicrobial treatments. Our group previously hypothesized that iron chelation therapy supports host defense during the early stages of bacterial infection by enhancing immunometabolic and inflammatory profiles in infected host cells (1). Mtb is now recognized as the world’s biggest infectious killer. In our current study, we show that the iron chelator DFX enhances glycolytic metabolism and boosts immune responses in a human macrophage model of early Mtb infection, and in cells stimulated with LPS (Figure 7). Mechanistically, our results indicate that DFX modulates the function of Mtb-infected human macrophages through the stabilization of HIF1α.

DFX has previously been shown to stabilize HIF1α in human renal Hep3B cells (37), in murine RAW 264.7 macrophages (36), in rabbit alveolar macrophages and in hMDMs (35). As HIF1α is a well-established master regulator of glycolytic metabolism and host defense during Mtb infection (1, 14), and the activity of HIF1α can be influenced by iron availability (53), we examined if DFX was able to enhance glycolytic metabolism in human macrophages infected with Mtb. We found that DFX significantly enhanced glycolytic metabolism at the transcript and cellular level in uninfected/unstimulated, LPS-stimulated and Mtb-infected human macrophages. To our knowledge, no previous study has shown that DFX induces glycolysis in a macrophage model of infection. It is also possible that the induction of glycolysis could partially be a result of a reduction in oxygen transport in the electron transport chain itself, due to low iron levels, resulting in an upregulation of glycolysis to compensate for the reduction in ATP generated by the electron transport chain (54). Therefore, the use of DFX, or other iron chelating agents, could prove beneficial in artificially ‘switching on’ glycolysis in infected immune cells. As iron loading can also negatively affect the bactericidal activity of specific anti-tuberculosis drugs (55), the use of iron chelators may also help to enhance the efficacy of these antimicrobials.

In addition to the assumed pathogenic role of IL10 in tuberculosis disease progression (43, 44), the protective roles of the pro-inflammatory cytokines TNFα and IL1β have been well documented by *in vivo* murine models (13, 34), and for IL1β in hMDMs (10). Our data show that DFX enhances both transcript and secreted protein levels of IL1β and TNFα in hMDMs infected with Mtb. DFX also enhanced *IL1*β transcript levels in hAMs infected with virulent Mtb. DFX can also promote IL1β release from LPS-stimulated hAMs from smokers and non-smokers (52). As DFX increased the secretion of both IL1β and TNFα, the opposite may be expected with the addition of exogenous iron. Indeed, the addition of iron significantly reduces TNFα, IL1α, IL1β and IL6 transcripts, along with TNFα protein levels, in J774 macrophages infected with Mtb H37Rv, an observation which is also accompanied with increased Mtb growth (42). We found DFX had no effect on IL10 expression levels in hMDMs stimulated with Mtb iH37Rv or Mtb H37Ra, however, we did find that DFX reduced IL10 protein levels in Mtb iH37Rv-stimulated hMDMs under hypoxia, highlighting potential clinical utility perhaps, as hypoxic lesions occur in pulmonary tuberculosis (56). Moreover, as IL10 has been implicated in the blockade of TNFα, IL1β and IL12 in a murine macrophage model of Mtb H37Rv infection, negating IL10 could support host defense (57). These results also suggest that DFX likely modulates IL10 function in macrophages independently of HIF1α.

IL1β shares an intrinsic relationship with both glycolytic metabolism and HIF1α, particularly during Mtb infection (14, 30). Using IL1β as the primary readout to examine the potential mechanism behind DFX, we found that DFX, at least in part, modulates cell function in Mtb-infected human macrophages through HIF1α. Although DFX induced glycolysis in uninfected/unstimulated, LPS-treated and Mtb-infected macrophages, the ability of DFX to modulate IL1β and TNFα production was specific only to LPS-treated and Mtb*-*infected cells. These disparities in the effect of DFX on metabolism and immune function are unsurprising, as DFX likely influences these two processes downstream of HIF1α independently. As discussed, DFX primarily influences metabolism through HIF1α stabilization resulting in downstream transcriptional and translational regulation of glycolytic metabolism. This process requires one signal, in this case HIF1α stabilization, which likely explains the induction of glycolysis in both uninfected and Mtb-infected macrophages. As the induction of IL1β secretion requires two stimuli; the lack of TLR and TNFαR engagement, and the absence of PAMPs and DAMPs in DFX-treated uninfected macrophages explains the lack of IL1β production. The addition of DFX to Mtb-infected macrophages enhances downstream HIF1α-induced expression of IL1β; we show enhanced HIF1α binding to the IL1β promoter in the presence of DFX and blockade of HIF1α to this promoter negates the induction of IL1β expression in Mtb-infected macrophages treated with DFX.

Targeting HIF1α with PX-478 or α-KG failed to alter TNFα levels in Mtb-infected macrophages treated with DFX. Nevertheless, studies have characterized the link between HIF1α and TNFα in LPS-stimulated macrophages (58–60). Accordingly, because DFX stabilizes HIF1α in the current system, it is likely that DFX also enhances TNFα in Mtb-infected macrophages through the stabilization of HIF1α. Moreover, as NFκB is a well-known regulator of TNFα levels (61), enhanced expression of NFκB, as observed in this study, may also contribute to the elevated TNFα levels in DFX-treated Mtb-infected macrophages. Further mechanistic studies, however, are required to advance our knowledge on how DFX regulates TNFα in DFX-treated Mtb-infected macrophages. It is also possible that IL1β and TNFα function in a positive feedback loop and promote further activity of HIF1α (62, 63). This further highlights the complex molecular intricacies that exist within Mtb-infected macrophages treated with DFX. For example, even though HIF1α deficiency is known to increase IL10 levels during Mtb infection (14), we also show that DFX reduces IL10 levels under hypoxic conditions. This implies that in addition to HIF1α, other molecular mediators may play a role in how DFX alters the metabolism and function of these macrophages. Such cellular mediators should be identified in future studies examining how DFX, and other iron chelators, function in macrophages to elicit changes in macrophage metabolism and function.

Even though Mtb infection has been reported to induce a shift from OXPHOS to glycolysis in hMDMs and hAMs (10), it has also been reported to have the opposite effect in hMDMs, that is, to decelerate bioenergetic profiles (64). However, these studies and others agree on the importance of glycolytic metabolism in host defense against Mtb infection. It is also likely that some deviations in these studies can be attributed to ontological differences that functionally separate tissue resident AMs from infiltrating MDMs, as demonstrated in murine models (11). Moreover, although DFX treatment increased metabolic transcripts comparably in both unstimulated hMDMs and hAMs in the current study, future studies delineating host metabolism in tissue resident hAMs and infiltrating hMDMs are essential. Additionally, as hAMs are predominantly considered to be tolerogenic, or M2-like, our data illustrating that both the immunometabolic and proinflammatory immune response of both hAMs and hMDMs can be supported with DFX could be valuable. In keeping with emerging findings, murine AMs can also be trained to be more proinflammatory (65). Thus the ability of a HDT to transiently switch on metabolism during early Mtb-infection could hold valuable therapeutic potential. In fact, we observe that Mtb-infected macrophages exhibited reduced ability to increase their glycolytic metabolism compared to uninfected macrophages when their ability to produce ATP through ATP synthase was inhibited; however, DFX conferred these macrophages with the ability to boost their glycolytic capacity. In the current study we observe that DFX can significantly alter the expression of genes (*RPIA*, *CPT1A* and *GLS*) involved in the pentose phosphate pathway, fatty acid metabolism and glutamine metabolism in hMDMs stimulated with Mtb iH37Rv, however, we could not recapitulate these findings in hMDMs infected with live Mtb. Regardless of the effect of DFX on these alternative metabolic pathways, DFX was still able to specifically enhance glycolytic metabolism in hMDMs infected with dead and live Mtb strains, and in hAMs infected with live virulent Mtb. This further highlights the potential clinical utility of DFX as an immunometabolic HDT against Mtb infection, and other infectious diseases.

The current study is not without its limitations. Live Mtb elicits changes in host cell function that may not be reflected in our model using iH37Rv, for example, through secreted virulence factors and complex immune evasion strategies. As iron chelators directly affects Mtb proliferation and viability, the use of the iH37Rv strain allowed us to assess the impact of DFX on hMDMs metabolism and function without DFX impacting live Mtb. Indeed, we also show that DFX promotes glycolysis in resting and LPS-stimulated cells reflecting DFX’s potential as a potential therapeutic immunomodulator. Moreover, as hAMs are only available in limited amounts for *in vitro* studies, this can significantly hinder highly detailed molecular analyses investigating host-Mtb interactions in these cells (50). As DFX-induced increases in IL1β could not be abrogated by 2-DG, further studies will be necessary to uncover how DFX-induced increases in glycolysis specifically benefit the host macrophage response. Importantly, such studies should also demonstrate a direct link between iron levels and metabolic responses in primary human macrophages. As this is the first time a study has demonstrated DFX’s ability to enhance glycolytic metabolism in uninfected and Mtb-infected human macrophages, the utility of DFX, and other iron chelators, needs to be validated in other primary cells *in vitro* and in suitable pre-clinical *in vivo* models to properly determine, comprehend, and uncover the untapped potential they could hold for therapeutic purposes. Accordingly, the use of DFX is also a potential limitation as some iron chelators are known to exhibit opposite effects in the same experimental model, as previously shown in deferasirox and DFX-treated neutrophils (66, 67). Iron chelators also possess different membrane permeability characteristics. DFX possesses low membrane permeability whereas others such as sibyllin and deferiprone exhibit higher membrane permeability qualities, which may also affect how they influence cell metabolism and function.

Our data highlights an emerging prospect in the metabolism field that could be therapeutically exploited. The dual effects of DFX as a glycolytic switch and as a means to support cellular function could have therapeutic implications for other infectious and non-infectious diseases. In addition to its effects on macrophage function, DFX has also been shown to contribute to the formation of neutrophil extracellular trap (NET) formation in human blood-derived neutrophils and offers a promising option for the manipulation of NET formation (67). As iron loading can also negatively affect the bactericidal activity of certain anti-tuberculosis drugs (55, 68), DFX also has the potential to improve the efficacy of antimicrobials which currently require many months of administration to eradicate tuberculosis infection. Therefore, in addition to its ability to enhance glycolytic metabolism and cytokine secretion, we hypothesize that DFX will have multifaceted effects on immune function and holds potential to be utilized as an immunometabolic HDT to boost the early host immune response to bacterial infection.

## Data Availability Statement

All datasets generated for this study are included in the article/Supplementary Material.

## Ethics Statement

The studies involving human participants were reviewed and approved by the St. James’s Hospital/Tallaght University Hospital Joint Research Ethics Committee, Dublin, Ireland. The patients/participants provided their written informed consent to participate in this study.

## Author Contributions

JP: conceptualization, data curation, formal analysis, software, visualization, and writing original draft. JK and JP: funding acquisition. JP, KM, CK, KG, DC, SB, SO’L, ST, and CÓ: investigations. JP, JK, KM, DC, SB, LO’N, MJO’S, and MPO’S: methodology. JP, KG, JK, and MPO’S: project administration. KG, DC, SB, JK, LO’N, and MJO’S: resources. All authors: validation, writing, reviewing and editing.

## Conflict of Interest

The authors declare that the research was conducted in the absence of any commercial or financial relationships that could be construed as a potential conflict of interest.

## Supplementary Material

FIGURE S1 Timing optimization of DFX treatment in uninfected and Mtb-infected hMDMs. hMDMs were infected with Mtb H37Ra and treated with DFX (100 μM) at the times indicated. 24 h post infection supernatants were harvested and **(A)** IL1β (*n* = 6) and **(B)** TNFα (*n* = 6) were quantified by ELISA. **(C)** The phagocytic ability of hMDMs treated with DFX, was assessed using auramine stained Mtb iH37Rv by fluorescent microscopy (*n* = 3). **(D)** The effect of DFX on the viability of Mtb iH37Rv-stimulated hMDMs was also assessed using a propidium iodide based cell exclusion assay (*n* = 4). Bars denote mean ± SEM. ^∗^*P* < 0.05 and ^∗∗^*P* < 0.01 (**A,B:** Two-way ANOVA with Bonferroni *post hoc* tests; **C,D:** Friedman ANOVA with Dunn’s multiple comparison test).

FIGURE S2 DFX enhances glycolytic metabolism in hMDMs stimulated with LPS. hMDMs, differentiated from PBMCs isolated from healthy blood donors, were stimulated with LPS (100 ng/mL) for 3 h, washed, and treated with DFX (100 μM). 24 h post infection transcript levels of **(A)** *PFKFB3* (*n* = 10), **(B)** *GAPDH* (*n* = 10) **(C)**, *PKM2* (*n* = 10) (representing glycolysis), and **(D)** *ATP5B* (*n* = 10) (representing oxidative phosphorylation) were determined by RT-qPCR. The effect of DFX on real-time baseline **(E)** ECAR and **(F)** OCR profiles, representing glycolysis and oxidative phosphorylation, respectively, was determined utilizing Seahorse extracellular flux assays in hMDMs 24 h post LPS stimulation (*n* = 7). **(G)** The ECAR:OCR ratio was generated to measure the reliance of one metabolic pathway over another (*n* = 7). **(H)** The immunometabolic shift due to DFX treatment is illustrated by the metabolic phenogram in LPS-stimulated hMDMs. **(I)** After treating LPS-stimulated hMDMs with the ATP synthase inhibitor oligomycin (1 μM), the ability of DFX to restore glycolytic capacity was examined in these cells (*n* = 6). Bars denote mean ± SEM. ^∗^*P* < 0.05, ^∗∗^*P* < 0.01, and ^∗∗∗^*P* < 0.001 (Two-way ANOVA with Bonferroni *post hoc* tests).

FIGURE S3 DFX differentially affects the pentose phosphate pathway, fatty acid metabolism, glutamine metabolism and tryptophan metabolism, but only in hMDMs stimulated with Mtb iH37Rv. hMDMs, differentiated from PBMCs isolated from healthy blood donors, were stimulated with Mtb iH37Rv or infected with Mtb H37Ra for 3 h, washed to remove unphagocytosed Mtb, and were treated with DFX (100 μM). **(A)** *G6PD* and **(B)** *RPIA* transcript levels (representing the oxidative and non-oxidative pathways of the pentose phosphate pathway, respectively), **(C)** *CPT1A* and **(D)** *FASN* transcript levels (representing fatty acid oxidation and fatty acid synthesis, respectively) and **(E)** *GLS* and **(F)** *IDO1* transcript levels (representing glutamine and tryptophan metabolism, respectively) were assessed by RT-qPCR (*n* = 5). Bars denote mean ± SEM. ^∗^*P* < 0.05 and ^∗∗^*P* < 0.01 (Two-way ANOVA with Bonferroni *post hoc* tests).

FIGURE S4 DFX reduces *CPT1A* transcript levels, without affecting *G6PD*, *RPIA*, *FASN*, *GLS* or *IDO1* gene expression in unstimulated and LPS-stimulated hMDMs. hMDMs, differentiated from PBMCs isolated from healthy blood donors, were stimulated with LPS (100 ng/mL) for 3 h, washed, and were treated with DFX (100 μM). **(A)** *G6PD* and **(B)** *RPIA* transcript levels (representing the oxidative and non-oxidative pathways of the pentose phosphate pathway, respectively), **(C)** *CPT1A* and **(D)** *FASN* transcript levels (representing fatty acid oxidation and fatty acid synthesis, respectively) and **(E)** *GLS* and **(F)** *IDO1* transcript levels (representing glutamine and tryptophan metabolism, respectively) were assessed by RT-qPCR (*n* = 10). Bars denote mean ± SEM. ^∗^*P* < 0.05 and ^∗∗∗^*P* < 0.001 (Two-way ANOVA with Bonferroni *post hoc* tests).

FIGURE S5 DFX supports immune function by enhancing transcript and protein levels of CXCL8 in hMDMs infected with Mtb. hMDMs, differentiated from PBMCs isolated from healthy blood donors, were stimulated with Mtb iH37Rv or infected with Mtb H37Ra for 3 h, washed to remove unphagocytosed Mtb, and were treated with DFX (100 μM). 24 h post infection transcript (**A,C,E**; *n* = 5) and protein (**B,D,F**; *n* = 5) levels of CCL5 **(A,B)**, CXCL8 **(C,D)** and CXCL10 **(E,F)** were quantified by RT-qPCR and ELISA. Bars denote mean ± SEM. ^∗^*P* < 0.05, ^∗∗^*P* < 0.01, and ^∗∗∗^*P* < 0.001 (Two-way ANOVA with Bonferroni *post hoc* tests).

FIGURE S6 DFX enhances *NF*κ*B* and *IL12a* transcript levels in hMDMs stimulated with Mtb iH37Rv. hMDMs, differentiated from PBMCs isolated from healthy blood donors, were stimulated with Mtb iH37Rv or infected with Mtb H37Ra for 3 h, washed to remove unphagocytosed Mtb, and were treated with DFX (100 μM). 24 h post infection, RT-qPCR was employed to assess transcript levels of **(A)** *NF*κ*B*, **(B)** *IL12a*, and **(C)** *IL18* (*n* = 5). Bars denote mean ± SEM. ^∗^*P* < 0.05 and ^∗∗∗^*P* < 0.001 (Two-way ANOVA with Bonferroni *post hoc* tests).

FIGURE S7 DFX differentially affects transcript and protein levels of IL1β, TNFα and IL10 in hMDMs stimulated with LPS. hMDMs, differentiated from PBMCs isolated from healthy blood donors, were stimulated with LPS (100 ng/mL) and treated with DFX 3 h later (100 μM). 24 h post LPS stimulation transcript (**A–C**; *n* = 10) and protein (**D–F**; *n* = 5–23) levels of IL1β **(A,D)**, TNFα **(B,E),** and IL10 **(C,F)** were quantified by RT-qPCR and ELISA. Bars denote mean ± SEM. ^∗^*P* < 0.05 and ^∗∗^*P* < 0.01 (Two-way ANOVA with Bonferroni *post hoc* tests).

FIGURE S8 DFX supports immune function by enhancing transcript and protein levels of CXCL8 in hMDMs stimulated with LPS. hMDMs, differentiated from PBMCs isolated from healthy blood donors, were stimulated with LPS (100 ng/mL) and treated with DFX 3 h later (100 μM). 24 h post LPS stimulation transcript (**A–C**; *n* = 10) and protein (**D–F**; *n* = 11) levels of CCL5 **(A,D)**, CXCL8 **(B,E)** and CXCL10 **(C,F)** were quantified by RT-qPCR and ELISA. Bars denote mean ± SEM. ^∗∗∗^*P* < 0.001 (Two-way ANOVA with Bonferroni *post hoc* tests).

FIGURE S9 DFX treatment fails to modulate secreted levels of TNFα in Mtb-infected hMDMs through HIF1α. hMDMs, differentiated from PBMCs isolated from healthy blood donors, were stimulated with Mtb iH37Rv or infected with Mtb H37Ra for 3 h, washed to remove unphagocytosed Mtb, and were treated with DFX (100 μM). **(A)** Optical density Western blot analysis of HIF1α protein levels normalized to β-actin levels (*n* = 3) (Image J). **(B)** We also examined if targeting HIF1α transcription/translation with PX-478 (25 μM) or if **(C)** blocking HIF1α-IL1β promotor binding with TFMB α-KG (1mM) could alter TNFα protein levels in Mtb H37Ra*-*infected hMDMs (*n* = 5) (DMSO concentration: 0.2%). **(D)** Using ChIP-qPCR analyses, we explored if DFX treatment could enhance HIF1α-IL1β promotor binding in unstimulated and Mtb iH37Rv*-*stimulated hMDMs 8 h post infection (*n* = 3). **(E,F)** Mtb H37Ra-infected hMDMs treated with DFX exhibited reduced CFUs by 25% 72 h post infection (*n* = 4). Bars denote mean ± SEM. ***P* < 0.01 (Two-way ANOVA with Bonferroni *post hoc* tests).
