# Supplementary figures and images for: Desferrioxamine Supports Metabolic Function in Primary Human Macrophages Infected With Mycobacterium tuberculosis
Source: Front Immunol. 2020 May 13;11:836. doi: 10.3389/fimmu.2020.00836 (PMC7237728; doi:10.3389/fimmu.2020.00836)

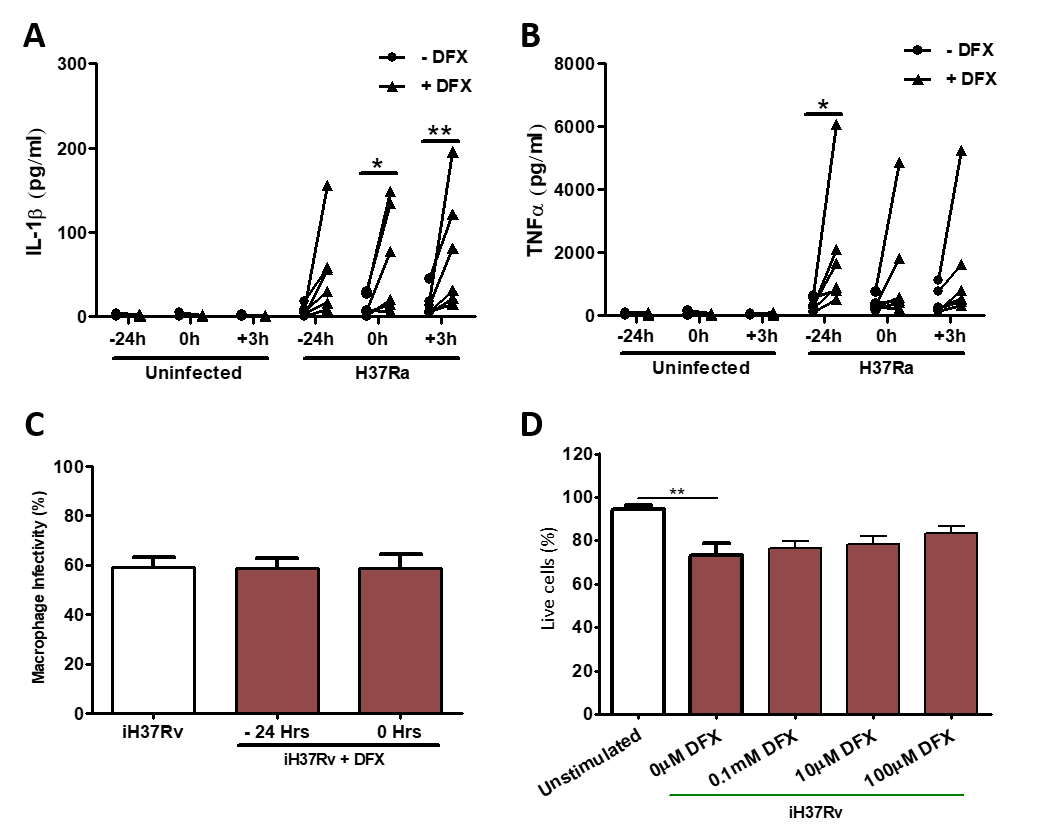

Supplement: FIGURE S1 — Timing optimization of DFX treatment in uninfected and Mtb-infected hMDMs. hMDMs were infected with Mtb H37Ra and treated with DFX (100 μM) at the times indicated. 24 h post infection supernatants were harvested and (A) IL1β (n = 6) and (B) TNFα (n = 6) were quantified by ELISA. (C) The phagocytic ability of hMDMs treated with DFX, was assessed using auramine stained Mtb iH37Rv by fluorescent microscopy (n = 3). (D) The effect of DFX on the viability of Mtb iH37Rv-stimulated hMDMs was also assessed using a propidium iodide based cell exclusion assay (n = 4). Bars denote mean ± SEM. ∗P < 0.05 and ∗∗P < 0.01 (A,B: Two-way ANOVA with Bonferroni post hoc tests; C,D: Friedman ANOVA with Dunn’s multiple comparison test). [file Image_1.TIF]

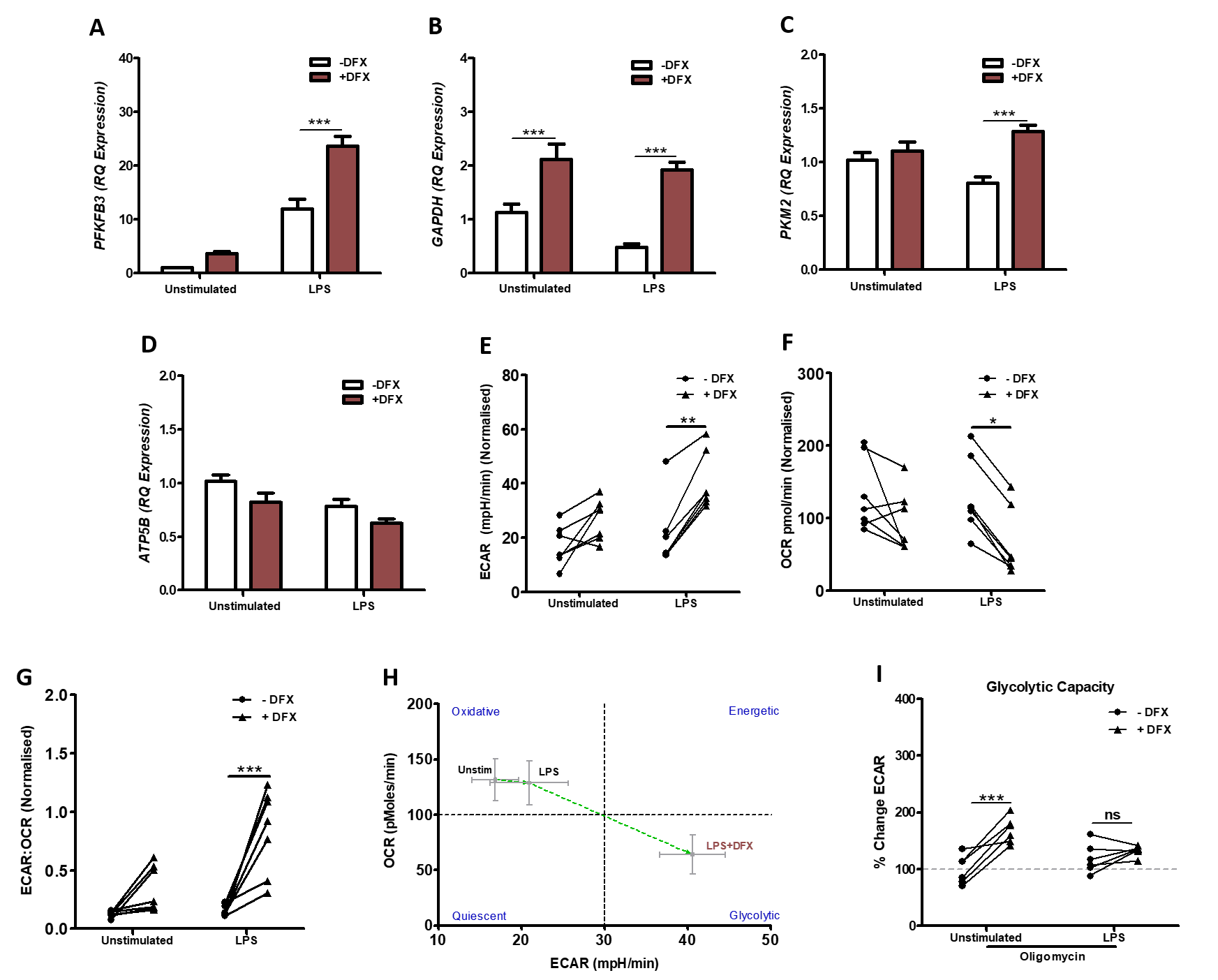

Supplement: FIGURE S2 — DFX enhances glycolytic metabolism in hMDMs stimulated with LPS. hMDMs, differentiated from PBMCs isolated from healthy blood donors, were stimulated with LPS (100 ng/mL) for 3 h, washed, and treated with DFX (100 μM). 24 h post infection transcript levels of (A) PFKFB3 (n = 10), (B) GAPDH (n = 10) (C), PKM2 (n = 10) (representing glycolysis), and (D) ATP5B (n = 10) (representing oxidative phosphorylation) were determined by RT-qPCR. The effect of DFX on real-time baseline (E) ECAR and (F) OCR profiles, representing glycolysis and oxidative phosphorylation, respectively, was determined utilizing Seahorse extracellular flux assays in hMDMs 24 h post LPS stimulation (n = 7). (G) The ECAR:OCR ratio was generated to measure the reliance of one metabolic pathway over another (n = 7). (H) The immunometabolic shift due to DFX treatment is illustrated by the metabolic phenogram in LPS-stimulated hMDMs. (I) After treating LPS-stimulated hMDMs with the ATP synthase inhibitor oligomycin (1 μM), the ability of DFX to restore glycolytic capacity was examined in these cells (n = 6). Bars denote mean ± SEM. ∗P < 0.05, ∗∗P < 0.01, and ∗∗∗P < 0.001 (Two-way ANOVA with Bonferroni post hoc tests). [file Image_2.TIF]

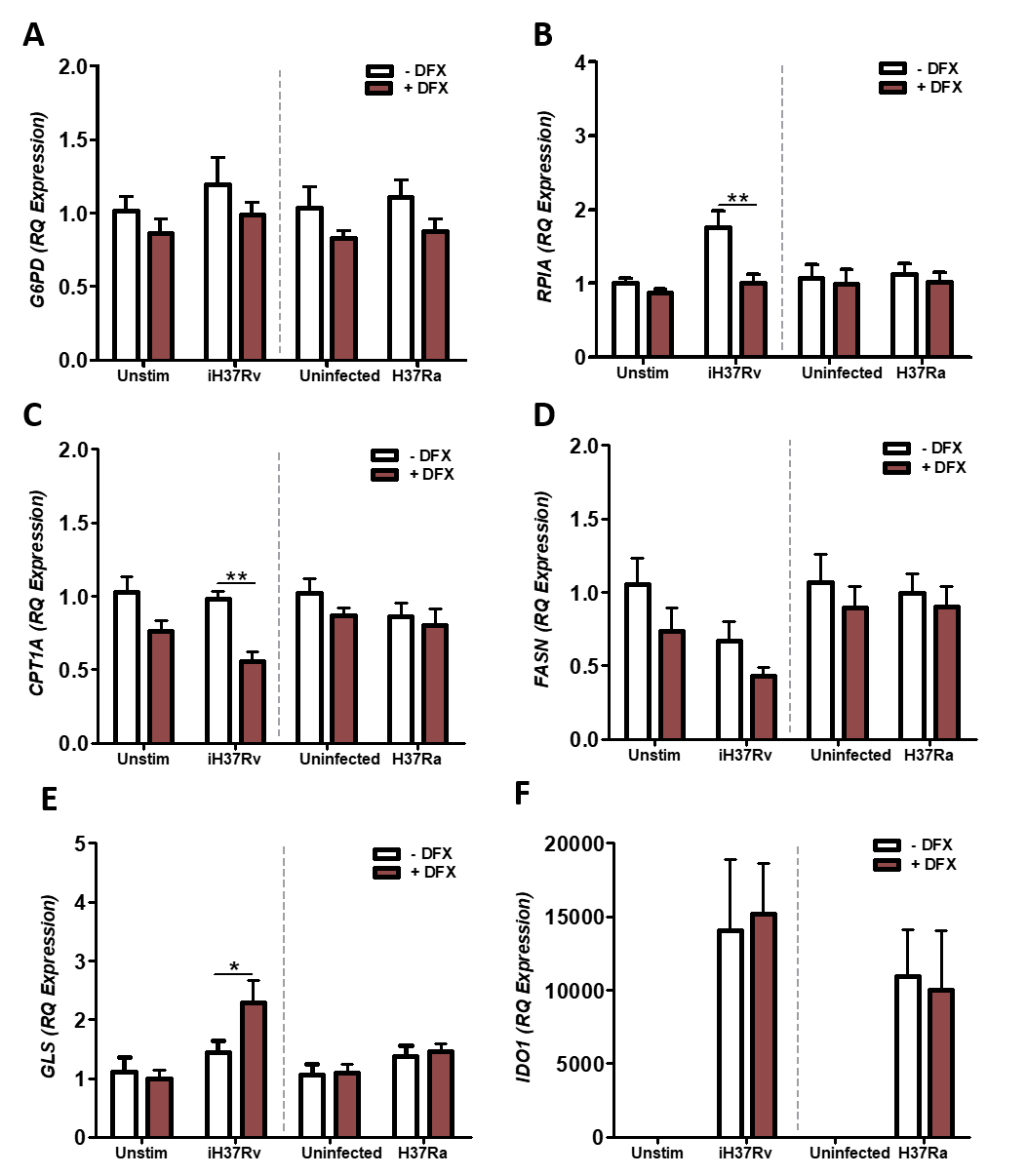

Supplement: FIGURE S3 — DFX differentially affects the pentose phosphate pathway, fatty acid metabolism, glutamine metabolism and tryptophan metabolism, but only in hMDMs stimulated with Mtb iH37Rv. hMDMs, differentiated from PBMCs isolated from healthy blood donors, were stimulated with Mtb iH37Rv or infected with Mtb H37Ra for 3 h, washed to remove unphagocytosed Mtb, and were treated with DFX (100 μM). (A) G6PD and (B) RPIA transcript levels (representing the oxidative and non-oxidative pathways of the pentose phosphate pathway, respectively), (C) CPT1A and (D) FASN transcript levels (representing fatty acid oxidation and fatty acid synthesis, respectively) and (E) GLS and (F) IDO1 transcript levels (representing glutamine and tryptophan metabolism, respectively) were assessed by RT-qPCR (n = 5). Bars denote mean ± SEM. ∗P < 0.05 and ∗∗P < 0.01 (Two-way ANOVA with Bonferroni post hoc tests). [file Image_3.TIF]

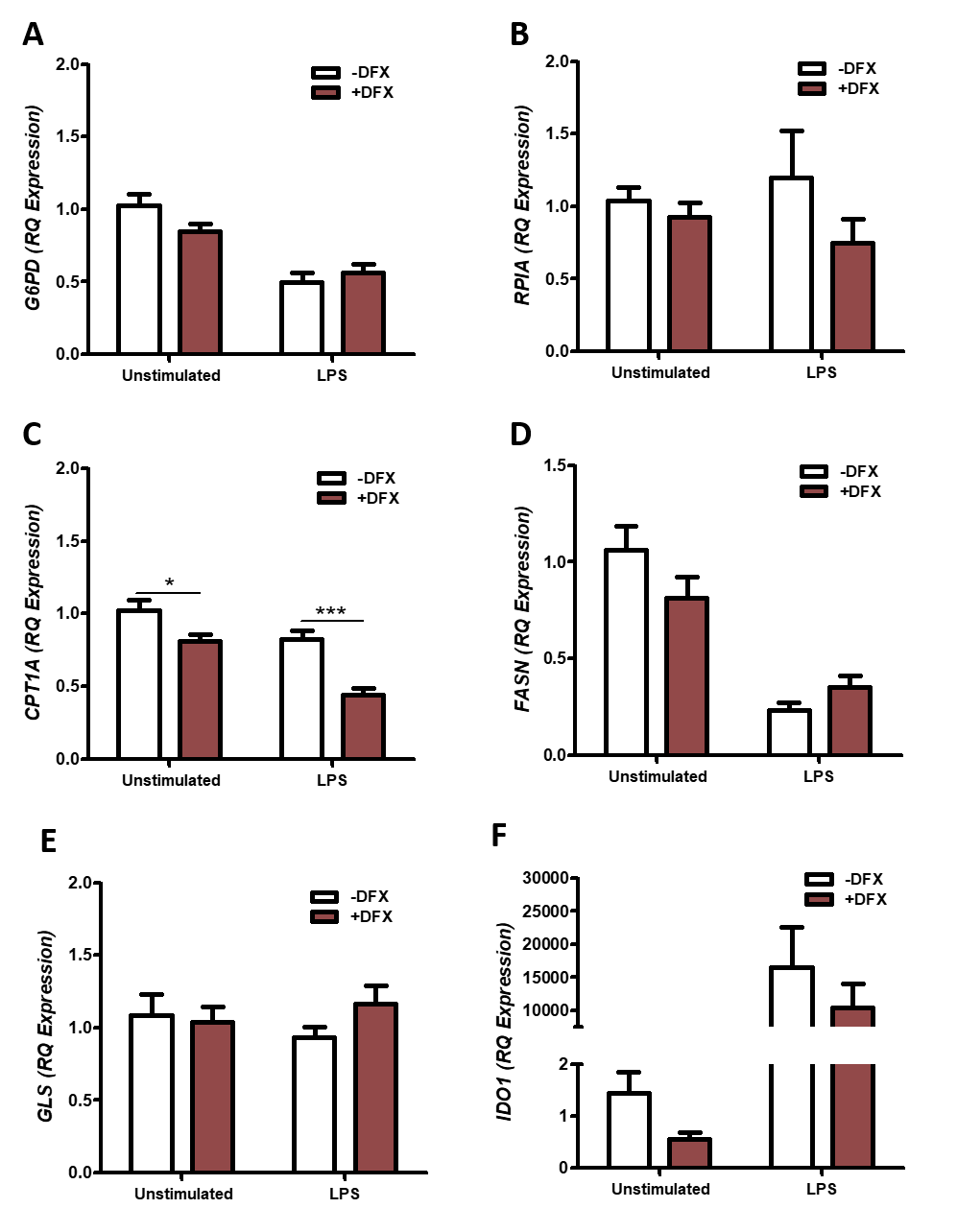

Supplement: FIGURE S4 — DFX reduces CPT1A transcript levels, without affecting G6PD, RPIA, FASN, GLS or IDO1 gene expression in unstimulated and LPS-stimulated hMDMs. hMDMs, differentiated from PBMCs isolated from healthy blood donors, were stimulated with LPS (100 ng/mL) for 3 h, washed, and were treated with DFX (100 μM). (A) G6PD and (B) RPIA transcript levels (representing the oxidative and non-oxidative pathways of the pentose phosphate pathway, respectively), (C) CPT1A and (D) FASN transcript levels (representing fatty acid oxidation and fatty acid synthesis, respectively) and (E) GLS and (F) IDO1 transcript levels (representing glutamine and tryptophan metabolism, respectively) were assessed by RT-qPCR (n = 10). Bars denote mean ± SEM. ∗P < 0.05 and ∗∗∗P < 0.001 (Two-way ANOVA with Bonferroni post hoc tests). [file Image_4.TIF]

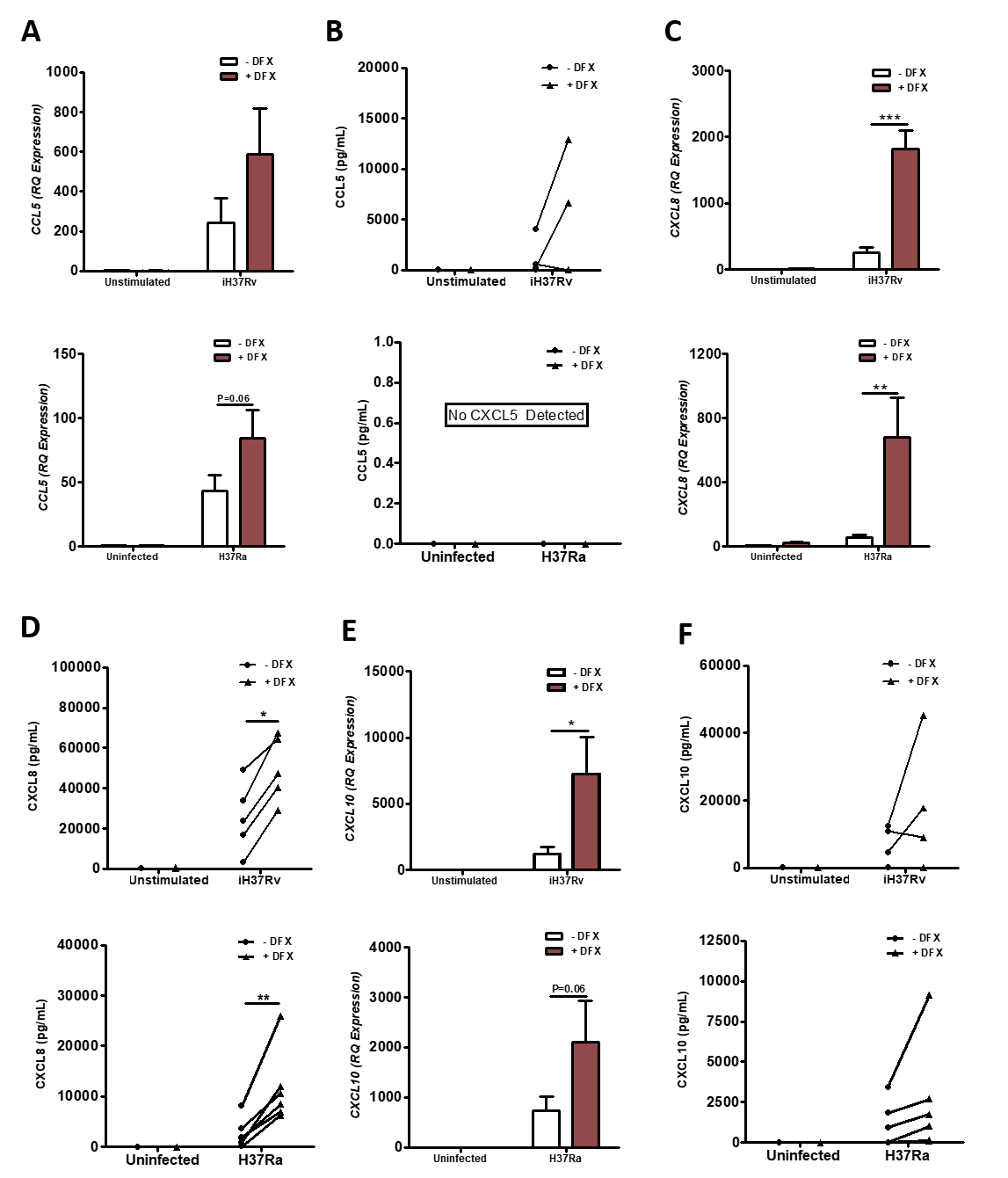

Supplement: FIGURE S5 — DFX supports immune function by enhancing transcript and protein levels of CXCL8 in hMDMs infected with Mtb. hMDMs, differentiated from PBMCs isolated from healthy blood donors, were stimulated with Mtb iH37Rv or infected with Mtb H37Ra for 3 h, washed to remove unphagocytosed Mtb, and were treated with DFX (100 μM). 24 h post infection transcript (A,C,E; n = 5) and protein (B,D,F; n = 5) levels of CCL5 (A,B), CXCL8 (C,D) and CXCL10 (E,F) were quantified by RT-qPCR and ELISA. Bars denote mean ± SEM. ∗P < 0.05, ∗∗P < 0.01, and ∗∗∗P < 0.001 (Two-way ANOVA with Bonferroni post hoc tests). [file Image_5.TIF]

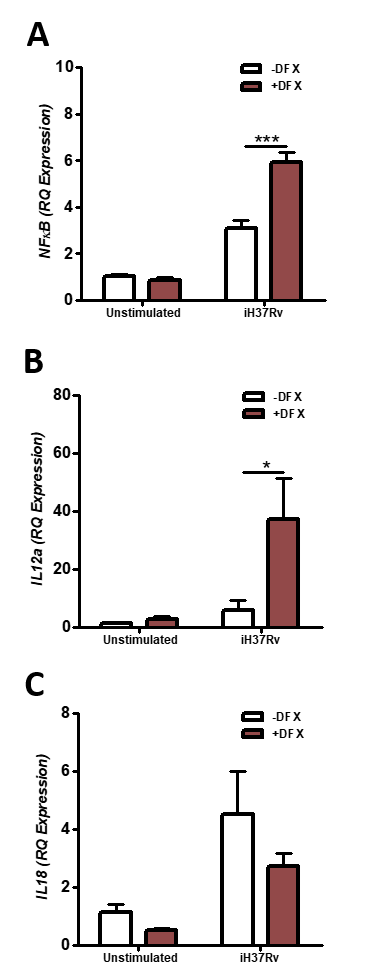

Supplement: FIGURE S6 — DFX enhances NFκB and IL12a transcript levels in hMDMs stimulated with Mtb iH37Rv. hMDMs, differentiated from PBMCs isolated from healthy blood donors, were stimulated with Mtb iH37Rv or infected with Mtb H37Ra for 3 h, washed to remove unphagocytosed Mtb, and were treated with DFX (100 μM). 24 h post infection, RT-qPCR was employed to assess transcript levels of (A) NFκB, (B) IL12a, and (C) IL18 (n = 5). Bars denote mean ± SEM. ∗P < 0.05 and ∗∗∗P < 0.001 (Two-way ANOVA with Bonferroni post hoc tests). [file Image_6.TIF]

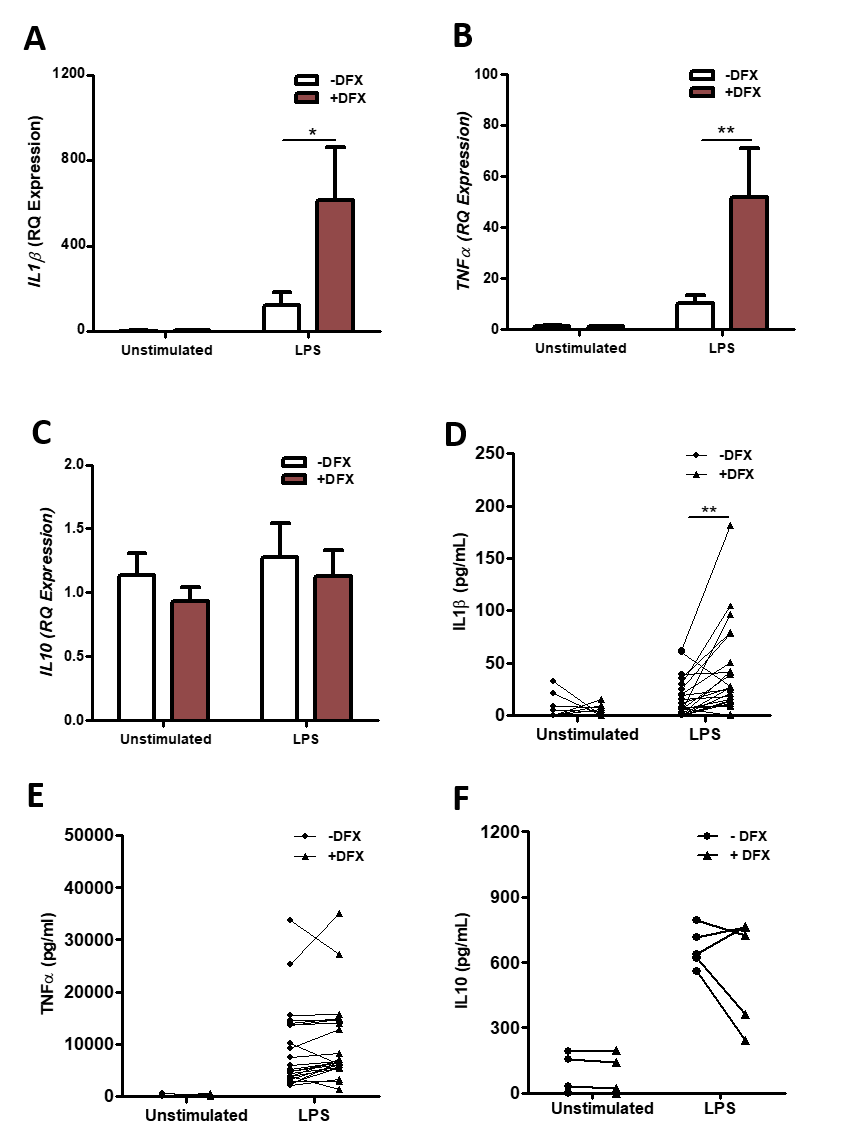

Supplement: FIGURE S7 — DFX differentially affects transcript and protein levels of IL1β, TNFα and IL10 in hMDMs stimulated with LPS. hMDMs, differentiated from PBMCs isolated from healthy blood donors, were stimulated with LPS (100 ng/mL) and treated with DFX 3 h later (100 μM). 24 h post LPS stimulation transcript (A–C; n = 10) and protein (D–F; n = 5–23) levels of IL1β (A,D), TNFα (B,E), and IL10 (C,F) were quantified by RT-qPCR and ELISA. Bars denote mean ± SEM. ∗P < 0.05 and ∗∗P < 0.01 (Two-way ANOVA with Bonferroni post hoc tests). [file Image_7.TIF]

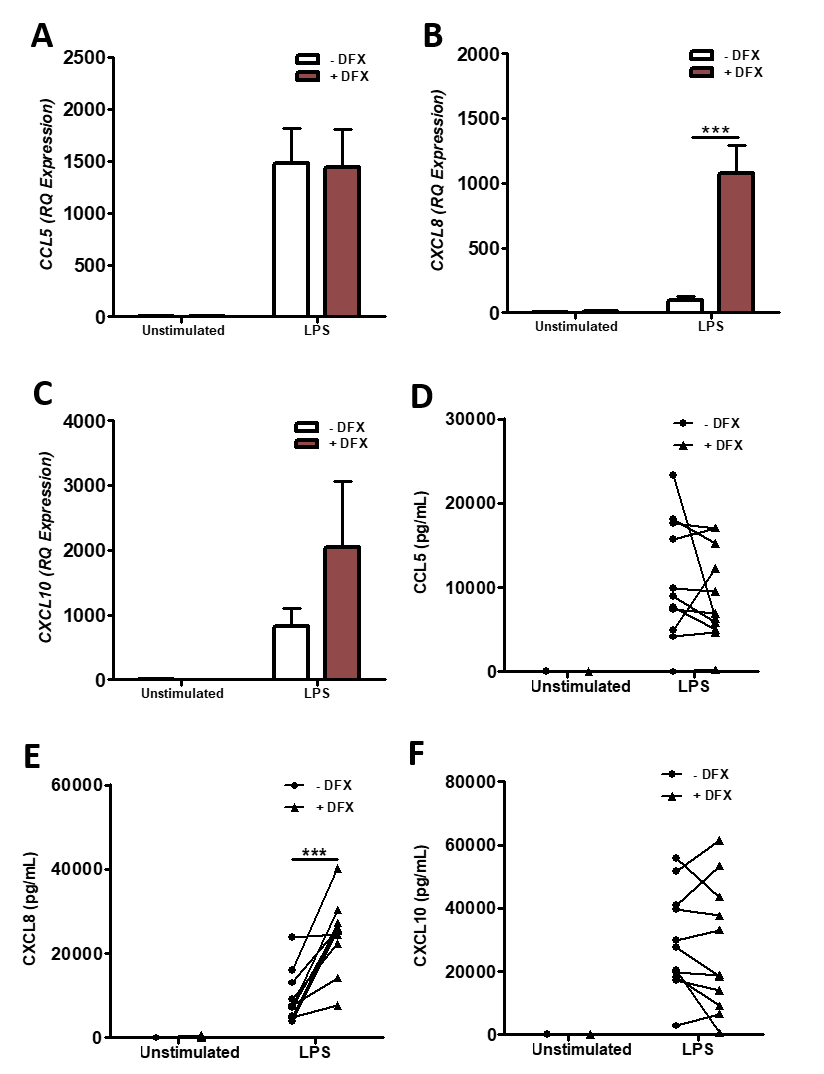

Supplement: FIGURE S8 — DFX supports immune function by enhancing transcript and protein levels of CXCL8 in hMDMs stimulated with LPS. hMDMs, differentiated from PBMCs isolated from healthy blood donors, were stimulated with LPS (100 ng/mL) and treated with DFX 3 h later (100 μM). 24 h post LPS stimulation transcript (A–C; n = 10) and protein (D–F; n = 11) levels of CCL5 (A,D), CXCL8 (B,E) and CXCL10 (C,F) were quantified by RT-qPCR and ELISA. Bars denote mean ± SEM. ∗∗∗P < 0.001 (Two-way ANOVA with Bonferroni post hoc tests). [file Image_8.TIF]

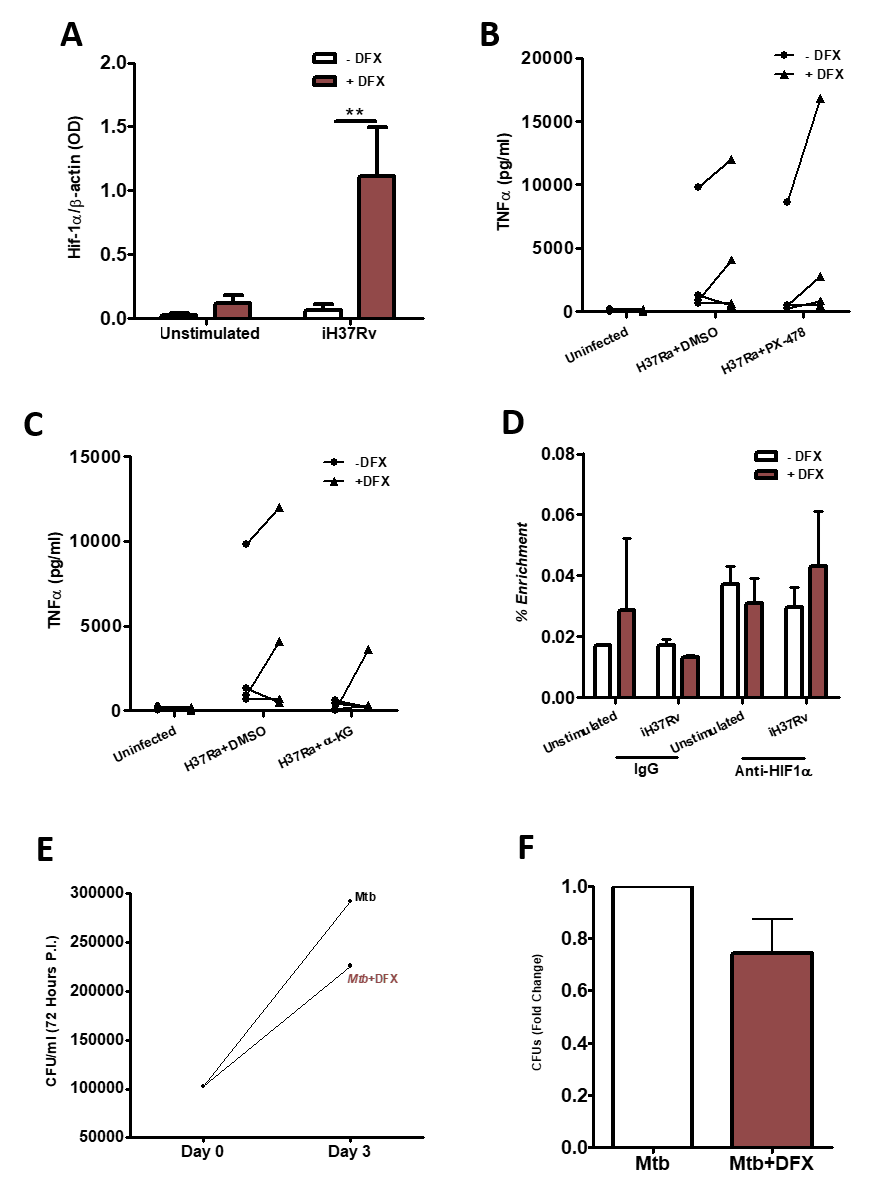

Supplement: FIGURE S9 — DFX treatment fails to modulate secreted levels of TNFα in Mtb-infected hMDMs through HIF1α. hMDMs, differentiated from PBMCs isolated from healthy blood donors, were stimulated with Mtb iH37Rv or infected with Mtb H37Ra for 3 h, washed to remove unphagocytosed Mtb, and were treated with DFX (100 μM). (A) Optical density Western blot analysis of HIF1α protein levels normalized to β-actin levels (n = 3) (Image J). (B) We also examined if targeting HIF1α transcription/translation with PX-478 (25 μM) or if (C) blocking HIF1α-IL1β promotor binding with TFMB α-KG (1mM) could alter TNFα protein levels in Mtb H37Ra-infected hMDMs (n = 5) (DMSO concentration: 0.2%). (D) Using ChIP-qPCR analyses, we explored if DFX treatment could enhance HIF1α-IL1β promotor binding in unstimulated and Mtb iH37Rv-stimulated hMDMs 8 h post infection (n = 3). (E,F) Mtb H37Ra-infected hMDMs treated with DFX exhibited reduced CFUs by 25% 72 h post infection (n = 4). Bars denote mean ± SEM. **P < 0.01 (Two-way ANOVA with Bonferroni post hoc tests). [file Image_9.TIF]
