# Supplementary material for: Desferrioxamine Supports Metabolic Function in Primary Human Macrophages Infected With Mycobacterium tuberculosis
Source: Front Immunol. 2020 May 13;11:836. doi: 10.3389/fimmu.2020.00836 (PMC7237728; doi:10.3389/fimmu.2020.00836)
Supplement: Supplementary file 10 [file Data_Sheet_1.docx]

**List of Reagents in Manuscript**

| ***REAGENT or RESOURCE*** | ***SOURCE*** | ***IDENTIFIER*** |
| --- | --- | --- |
| *Antibodies* | | |
| Western: Mouse Anti-Human HIF1α IgG1 | BD Biosciences | 610958 |
| Western: Mouse Anti-β-Actin antibody IgG2a | Sigma | A5316 (RRID: AB-476743) |
| Western: Goat anti-Mouse IgG IgG (Secondary) | Millipore | AP124P |
| ChIP: Rabbit anti-HIF-1α aa432-528 (ChIP Grade) | Abcam | ab2185 |
| Bacterial and Virus Strains | | |
| Irradiated H37Rv *Mtb* Strain (iH37Rv) | BEI Resources | NR-14819 |
| Avirulent H37Ra *Mtb* Strain (H37Ra) | ATCC | 25177 |
| Virulent H37Rv *Mtb* Strain (H37Rv) | ATCC | 25618 |
| Chemicals, Peptides, and Recombinant Proteins | | |
| Deferoxamine mesylate salt | Sigma Aldrich | D9533 |
| Lipopolysaccharide from E.coli O55:B5 | Sigma Aldrich | L5418 |
| Crystal Violet | Sigma Aldrich | C0775 |
| 2-Deoxy-D-Glucose | Sigma Aldrich | D6134 |
| Glutaraldehyde | Sigma Aldrich | G7651 |
| Dulbecco''s Phosphate Buffered Saline | Sigma Aldrich | D8537 |
| Lymphoprep | Stemcell Technologies | 07861 |
| RPMI 1640 Medium, GlutaMAX™ Supplement | Bio-Sciences | 72400054 |
| Human Serum Type AB (Male) From Male AB | Sigma Aldrich | H4522 |
| Trypan Blue Solution 0.4% (w/v) | Fisher Scientific | 15393661 |
| Foetal Bovine Serum | Gibco | 10270106 |
| Fungizone | Gibco | 15290018 |
| Cefotaxime | Melford Biolaboratories | C0111 |
| Middlebrook ADC | Becton Dickinson | BD-211887 |
| Middlebrook OADC | Becton Dickinson | BD-211886 |
| Middlebrook 7H9 Broth | Becton Dickinson | BD-271310 |
| Middlebrook 7H9 Agar | Becton Dickinson | BD-262710 |
| Tween 80 | Sparks Lab Supplies | BD-231181 |
| L-asparagine | Sigma Aldrich | A4159 |
| Cycloheximide | Sigma Aldrich | C7698 |
| Paraformaldehyde | Sigma Aldrich | 158127 |
| Modified Auramine O | Scientific Device Laboratory | 345-250 |
| DAKO Fluorescence Mounting Medium | Cruinn Diagnostics | S302380-2 |
| Sterile Purified Water | Merck Millipore | 4.86505.1000 |
| Propidium Iodide Solution | Sigma Aldrich | P4864 |
| bisBenzimide H 33342 trihydrochloride | Sigma Aldrich | B2261 |
| bisBenzimide H 33258 trihydrochloride | Sigma Aldrich | B1155 |
| PBS Tablets | Bio-Sciences | 18912014 |
| Halt Protease Inhibitor Cocktail | Bio-Sciences | 78429 |
| Phosphatase Inhibitor Cocktail | Sigma Aldrich | P5726 |
| TaqMan Universal PCR Master Mix No UNG | Applied Biosystems | 4324018 |
| CAPS | Sigma Aldrich | C2632 |
| Trizma hydrochloride | Sigma Aldrich | T3253 |
| Triton X-100 | Sigma Aldrich | T9284 |
| Sodium dodecyl sulfate | Sigma Aldrich | L3771 |
| Tween 20 | Sigma Aldrich | P2287 |
| 1,4-Dithiothreitol | Sigma Aldrich | 10197777001 |
| Glycerol | Sparks Lab Supplies | BD-228220 |
| Sodium deoxycholate | Sigma Aldrich | D6750 |
| WesternBright ECL | MyBio | K-12045-D20 |
| PageRuler Prestained Protein Ladder | Fisher Scientific | 26616 |
| Bromophenol Blue | Sigma Aldrich | 114391 |
| Protease Inhibitor Cocktail (ChIP) | Sigma Aldrich | P8340 |
| Blue/Orange Loading Dye (6x) | Promega | G190A |
| RNase A (DNase and protease free) | Thermo Scientific | EN0531 |
| Protease K (Molecular Grade) | New England Biolabs | P8107S |
| SensiFAST Sybr Hi-Rox Mix (ChIP) | Medical Supply Company | BIO-92020 |
| Glycogen (ChIP) | Sigma Aldrich | G1767 |
| Glycine (ChIP) | Sigma Aldrich | G8898 |
| Formaldehyde (ChIP) | Sigma Aldrich | F8775 |
| Leupeptin | Sigma Aldrich | L2884 |
| Aprotenin | Sigma Aldrich | A6106 |
| Phenylmethylsulfonyl Fluoride (PMSF) | Sigma Aldrich | P7626 |
| Ultra Pure Phenol Chloroform (ChIP) | Invitrogen | 15593-049 |
| Ethanol | Sigma Aldrich | E7023 |
| Agarose | Sigma Aldrich | A9539 |
| *Critical Commercial Assays* | | |
| Human IL1β ELISA MAX Deluxe Kit | Medical Supply Company | 437006 |
| Human IL10 ELISA MAX Deluxe Kit | Medical Supply Company | 430606 |
| Human TNFα ELISA Kit | Bio-Sciences | 88-7346-77 |
| RevertAid First Strand cDNA Synthesis Kit | VWR | K1622 |
| RNeasy Plus Mini Kit | Qiagen | 74136 |
| Pierce BCA Protein Assay Kit | Pierce | 23227 |
| Seahorse XFe24 FluxPak | Agilent Technologies | 102340-100 |
| Seahorse XF Cell Mito Stress Test Kit | Agilent Technologies | 103015-100 |
| GeneRuler 1 kb Plus DNA Ladder | Thermo Scientific | SM1331 |
| Dynabeads | Invitrogen | 10001D |
| *Oligonucleotides* | | |
| IL1β Fwd (ChIP)- CACACATGAACGTAGCCGTC | Sigma Aldrich | N/A |
| IL1β Rev (ChIP)- TTCACTGGCGAGCTCAGGTA | Sigma Aldrich | N/A |
| 18S: | Applied Biosystems | Hs03003631_g1 |
| G6PD: | Applied Biosystems | Hs00166169_m1 |
| RPIA: | Applied Biosystems | Hs01107136_m1 |
| FASN: | Applied Biosystems | Hs01005622_m1 |
| CPT1A: | Applied Biosystems | Hs00912671_m1 |
| GLS: | Applied Biosystems | Hs01014020_m1 |
| IDO1: | Applied Biosystems | Hs00984148_m1 |
| IL18: | Applied Biosystems | Hs01038788_m1 |
| IL10: | Applied Biosystems | Hs00961622_m1 |
| IL1B: | Applied Biosystems | Hs01555410_m1 |
| TNFa: | Applied Biosystems | Hs00174128_m1 |
| IL12a: | Applied Biosystems | Hs01073447_m1 |
| GAPDH: | Applied Biosystems | Hs02786624_g1 |
| PKM2: | Applied Biosystems | Hs00761782_s1 |
| PFKFB3: | Applied Biosystems | Hs00998698_m1 |
| ATP5B: | Applied Biosystems | Hs00969569_m1 |
| *Software and Algorithms* | | |
| GraphPad Prism 5 | GraphPad Software | <https://www.graphpad.com/scientific-software/prism/> |
